# Peripheral Nervous System Genes Expressed in Central Neurons Induce Growth on Inhibitory Substrates

**DOI:** 10.1371/journal.pone.0038101

**Published:** 2012-06-06

**Authors:** William J. Buchser, Robin P. Smith, Jose R. Pardinas, Candace L. Haddox, Thomas Hutson, Lawrence Moon, Stanley R. Hoffman, John L. Bixby, Vance P. Lemmon

**Affiliations:** 1 Miami Project to Cure Paralysis, Departments of Pharmacology and Neurological Surgery, and Neuroscience Program, University of Miami, Miller School of Medicine, Miami, Florida, United States of America; 2 Egea Biosciences, La Jolla, California, United States of America; 3 Division of Rheumatology and Immunology, Medical University of South Carolina, Charleston, South Carolina, United States of America; 4 Wolfson Centre for Age-Related Diseases, King’s College London, London, United Kingdom; Alexander Flemming Biomedical Sciences Research Center, Greece

## Abstract

Trauma to the spinal cord and brain can result in irreparable loss of function. This failure of recovery is in part due to inhibition of axon regeneration by myelin and chondroitin sulfate proteoglycans (CSPGs). Peripheral nervous system (PNS) neurons exhibit increased regenerative ability compared to central nervous system neurons, even in the presence of inhibitory environments. Previously, we identified over a thousand genes differentially expressed in PNS neurons relative to CNS neurons. These genes represent intrinsic differences that may account for the PNS’s enhanced regenerative ability. Cerebellar neurons were transfected with cDNAs for each of these PNS genes to assess their ability to enhance neurite growth on inhibitory (CSPG) or permissive (laminin) substrates. Using high content analysis, we evaluated the phenotypic profile of each neuron to extract meaningful data for over 1100 genes. Several known growth associated proteins potentiated neurite growth on laminin. Most interestingly, novel genes were identified that promoted neurite growth on CSPGs (GPX3, EIF2B5, RBMX). Bioinformatic approaches also uncovered a number of novel gene families that altered neurite growth of CNS neurons.

## Introduction

Many animals have the ability to regenerate damaged neurons after injury. Unfortunately, mature neurons in the mammalian central nervous system (CNS) generally lack this ability [1]. Axonal loss owing to spinal cord injury, traumatic brain injury, or neurodegenerative disorders requires long distance axon growth for functional recovery. However, due to intrinsic and extrinsic factors that limit axon regeneration in the CNS, disability is usually permanent. In contrast, peripheral nervous system (PNS) neurons have the ability to regenerate their axons, and have been observed to respond to regeneration-promoting manipulations in the presence of inhibitory myelin and CSPGs even when CNS neurons do not [2].

Intrinsic differences underlie the differing growth ability of peripheral and central neurons. Davies et al. [3] showed that adult dorsal root ganglion (DRG) neurons were able to grow long axons across the myelin rich corpus callosum of adult animals. CNS neurons transplanted under similar conditions failed to grow axons [4] suggesting that differences between the neuronal cell types determines their axons’ ability grow through regions containing CNS myelin. Similarly, adult DRG neurons have been observed to grow into sites rich in inhibitory CSPGs much more effectively than CNS neurons [5].

Intrinsic factors also play a role in the inability of mature CNS neurons to regenerate axons after injury. Raisman et al. [6] showed that young (P6-8) entorhinal cortex explants could send long axons into mature (P12-15) dentate gyrus explants. By contrast, mature entorhinal cortex neurons were unable to grow axons onto young dentate gyrus. Similar conclusions emerge from studies of purified retinal ganglion cells (RGCs) cultured at low density in isolation. Axons elongated quickly in RGCs from embryonic (E21) rats but neurons from an animal just a few days older (P2), were unable to elongate their axons [7]. High content screens of genes differentially expressed during development have identified the KLF family of transcription factors as important regulators of axon growth, along with cytoskeletal components such as doublecortin and doublecortin-like kinase [8], [9]. Recently, the genes for phosphatase and tensin homolog (PTEN) and Suppressor of cytokine signaling 3 (SOCS3) were transgenically deleted from RGCs. After optic nerve crush, these animals showed extensive axonal regeneration by mature RGCs [10], [11], without any other manipulation. This strongly supports the idea that the intrinsic state of CNS neurons plays a significant role in their inability to regenerate axons.

Extrinsic factors also inhibit axon growth. After an insult to the spinal cord, axons are often damaged, causing the proximal portion to retract while the distal axon degenerates. Oligodendrocytes that previously ensheathed the axons in the spinal pathways also degenerate, releasing myelin debris into the injury site. Inflammatory cells and astrocytes migrate to the injury site and probably cause secondary damage [12]. Myelin components derived from injured oligodendrocytes inhibit CNS axon regeneration [13]. Unfortunately, knocking out several of the molecules underlying that inhibition (or their receptors) has not led to impressive recovery [14].

Another source of inhibitory molecules at the injury site is the glial scar, formed by reactive astrocytes that express chondroitin sulfate proteoglycans (CSPGs) and other inhibitory proteins [15]. CSPGs are highly inhibitory to neurite growth both *in-vitro* [16] and *in-vivo* [17]. Downstream pathways mediating CSPG axon growth inhibition in neurons are still poorly characterized, although receptor protein tyrosine phosphatases [18], the EGF receptor [19], and conventional PKC [2] have been implicated in neuronal responses to these signals. Interestingly, a PKC inhibitor (Gö6976), when applied *in-vivo* after spinal cord dorsal hemi-section, induced robust regeneration of dorsal column axons across the scar while the descending pathways of the corticospinal tract failed to regenerate. Chondroitinase, which degrades most of the glycosaminoglycan chains from the core CSPG protein, has also been used to decrease the inhibitory nature of the scar. This has achieved positive effects [20]. Thus both environmental and neuron-intrinsic characteristics must be understood to successfully promote regeneration of CNS axons.

One way to identify genes associated with increased regenerative capacity is by overexpression of candidate cDNAs. Candidate lists may be obtained by identifying genes differentially expressed among neurons with differing regenerative abilities [21]. As previously reported, we employed a subtraction cDNA library and microarray data to identify genes enriched in DRG (PNS) neurons compared to CNS neurons [22]. Here we report the testing of over 1100 genes preferentially expressed in PNS neurons in an unbiased, high content screen to characterize the functional effect of PNS gene overexpression in CNS neurons. In addition to identifying individual candidate genes for subsequent study, we have used this large data set to perform a systems-level analysis aimed at uncovering novel gene clusters and signaling pathways associated with axon growth.

## Results

### Identification of PNS Enriched Genes

We identified PNS enriched genes by subtractive hybridization of two cDNA libraries: postnatal day 8 (P8) mouse DRG and P8 mouse cerebellum [22]. From 2,016 cDNA clones, we obtained high quality sequence corresponding to 1,100 known genes (many of which were sequenced more than once).

PNS enriched genes identified in the subtraction library were initially validated two ways; by public microarray and quantitative PCR (QPCR; Figure S1). The subtraction library was complemented with an *in silico* subtraction of microarray data from laser capture micro-dissected DRG neurons and whole cerebellum. The combination of these data was used to construct the final list of DRG enriched genes [22].

A large cDNA library from the Mammalian Genome Collection [23] was queried for the presence of 1,381 genes in the final gene list. 889 of the genes were found in the library. This set of nonredundant genes was subjected to further analysis using the Allen Brain Atlas [24], to confirm their low cerebellar expression compared with other brain tissue (Figure S2). Because of the existence of both human and mouse clones (and occasional variant isoforms), we picked 1,300 clones representing these genes from the MGC collection to generate a “DRG library” (Source Data File).

### Inhibitory Growth Assay

**Figure 1:**
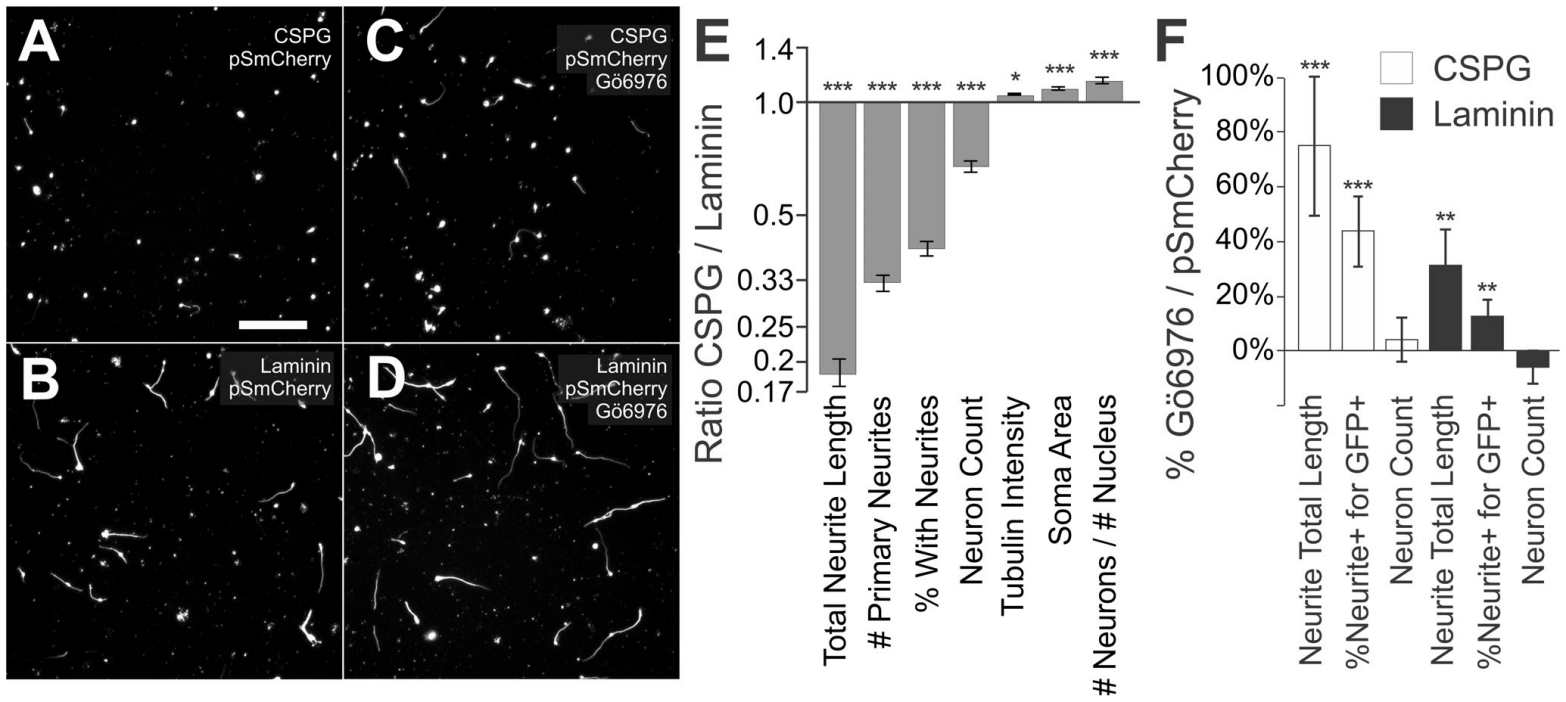
Cerebellar granule neurons are robustly inhibited by CSPGs and partially rescued by Gö6976. Dissociated postnatal cerebellar granule neurons (CGNs) were challenged in an inhibitory assay. ***A, B***, representative images of CGNs transfected with control plasmid pSport mCherry growing on CSPGs (**A**), and a permissive substrate, laminin (**B**). ***C,D***, the addition of the PKC inhibitor Gö6976 partially relieved CSPG inhibition (**C**), and potentiated laminin growth (**D**). ***E*** *,* Bar chart depicting ratios of seven parameters on CSPGs divided by laminin (with 95% confidence intervals). Growth on CSPGs led to large decreases in neurite length, the number of primary neurites, and the percentage of cells with neurites. Tubulin intensity and soma area were slightly increased. The neuron count was decreased on CSPGs, implying a deficiency in cell adhesion or survival. ***F***, Percent increase by Gö6976 was plotted with 95% confidence intervals on CSPGs (open bars), and on laminin (solid bars). Gö6976 had a strong positive effect on neurons growing on CSPGs, especially for neurite length and the percentage of neurons growing neurites. Neuron count and viability was unchanged. Statistics with un-normalized treatment averages, Mann-Whitney Test, *p< = 0.05, **p< = 0.01, ***p< = 0.0001. Scale bar 100 µm.

We hypothesized that DRG neurons selectively express regeneration-associated genes, which if overexpressed in CNS neurons could promote axon regeneration. We therefore tested the neurite growth promoting ability of each PNS gene in cerebellar granule neurons (CGNs). CGNs are widely used in the study of neurite growth inhibition [25], [26], [27], and to this end we developed an inhibitory growth assay. We challenged postnatal mouse CGNs to grow on an inhibitory substrate, comparing their neurite extension with control neurons grown on permissive substrates. We initially developed a neurite outgrowth assay using CNS myelin as an inhibitory substrate, which was used in a small preliminary screen (Table S1). Because this proved difficult to scale up for a full screen we used a mixture of chondroitin sulfate proteoglycans and laminin (referred to as simply CSPGs) as the inhibitory substrate and laminin alone as the permissive substrate [28]. Postnatal CGNs exhibited a normal bipolar morphology on laminin (Fig. 1B) and were strongly inhibited by CSPGs (Fig. 1A). Principle component analysis and strictly standardized mean difference (SSMD) analysis indicated that total neurite count and length were the most reliable feature relating to neurite growth of the dozen features reported by the Cellomics Neuronal Profiling Bio-application. The total neurite length for each neuron was reduced fivefold on CSPGs (p<10^−29^, Mann-Whitney U test [MWU]). The number of primary neurites and the percent of neurons that initiated neurite growth were each reduced about threefold on CSPGs compared to laminin (Fig. 1E). Growth inhibition by CSPGs was present at all basal levels of neurite outgrowth on laminin, which varied among experiments (Figure S3). Intensity of tubulin staining and the area of the soma were slightly but significantly increased on CSPGs (5% and 9% respectively, Fig. 1E). Dead neurons were easily distinguished by positive Hoechst signal with no accompanying tubulin positive soma. This parameter (the ratio of neurons per nucleus) is a rough measure of neuronal viability, and was increased slightly in CSPGs compared to laminin (Fig. 1E). Thus, CSPGs robustly inhibited neurite growth, reduced adhesion, and didn’t have a strong effect on viability.

The negative control for neurite outgrowth was transfection of mCherry [29] in the pSport CMV vector. The negative control and experimental genes were all in the same vector (pSport 6.0) with identical CMV promoters. For positive control, the pro-regenerative PKC inhibitor Gö6976 [2] was added in addition to the transfection of mCherry. For neurons growing on CSPGs (Fig. 1F, white bars), Gö6976 was able to increase the total length of the neurites by 70% and the percent of neurons initiating neurite growth by 40% (p<10^−8^ MWU), but had no effect on the number of neurons counted (p = 0.79 MWU), or the neuron/nucleus ratio (not shown, p = 0.18 MWU). Gö6976, as in [2], relieved the inhibition of the neurite growth on CSPGs by about 20%, but did not restore neurons to their full laminin growth potential. For laminin, neurite growth was also potentiated by Gö6976 (Fig. 1F, black bars). Therefore, we have developed an assay system in which postnatal mouse CGNs can be transfected and examined for neurite growth on CSPGs. Robust inhibition of neurite growth was observed on CSPGs and could be overcome by Gö6976.

### Functional Evaluation of 1000 PNS Genes in Cerebellar Neurons

From the list of differentially expressed genes (results of subtractive hybridization and microarray), we found many candidates for further study based on the literature. Details of those genes are discussed by [22]. However, of the 927 non-redundant genes, 9% had not been previously reported in PubMed (except in mammalian genome papers), 50% were the subject of twenty or fewer articles, and only 10% were mentioned in a hundred or more papers (as of May 2009). Instead of pursuing individual genes of interest based on the literature, we performed an unbiased phenotypic screen, overexpressing each gene from the DRG library in primary CGNs. We utilized High Content Screening (HCS) to carefully analyze the phenotypes of every accessible neuron in a treatment condition [30]. After quality control, we obtained data from a total of 1,132 cDNA clones in neurons from 348 different 96-well plates (∼13,000 total wells). 4,626,954 individual neurons were automatically traced and analyzed for over twenty different morphological parameters (nuclear, cell body, neurite, population parameters). All treatments were represented in three *internal* replicates, with an average of 899 individual neurons per gene (treatment) on CSPGs and 1,197 neurons per treatment on laminin across the three internal replicate wells. Two *experimental* replicates for each gene were performed with neurons from different mice on different days.

Although our goal was to identify genes that increase the neurite growth of neurons on inhibitory substrates, the screen also allowed for identification of a variety of other functional classes. Our assay was able to detect both increases and decreases in neurite growth, thus identifying activators and inhibitors. The use of two substrates enabled the detection of environmental-specific effects and cell health-related effects. Neurite initiation, a measure of the percent of neurons in a treatment condition that grew neurites, was a good measure of inhibition, since many neurons did not initiate growth on the inhibitory substrate. In contrast to highly polarized hippocampal neurons [30], which do not exhibit a strong correlation between number of primary neurites and overall neurite length, neurite parameters for CGNs in this study were highly correlated and grouped together, suggesting that CGN neurons and hippocampal neurons use different mechanisms to regulate neurite growth (Figure S5).

**Figure 2:**
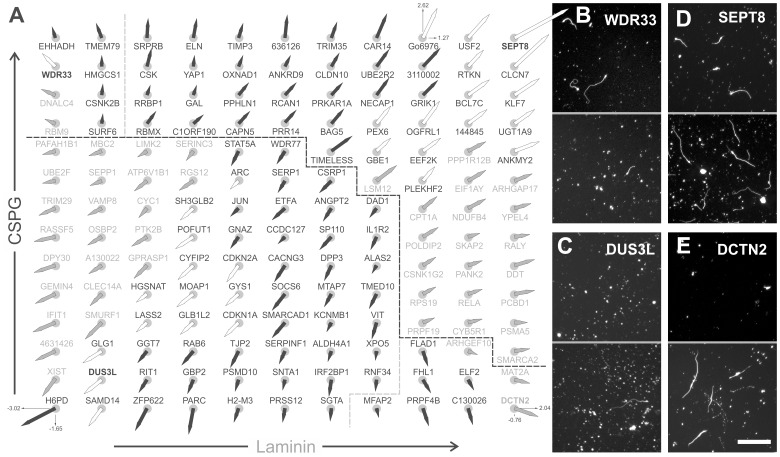
Significant morphological changes after peripheral gene expression in CGNs. ***A***, Vector grid of 154 genes from the screen, measuring neurite initiation on CSPG (vector points upward if growth was increased, downward if decreased) and laminin (vector points rightward if increased, leftward if decreased). Scale is the same for all genes and is indicated at the bottom corners (H6PD, DCTN2) and at the top right for the positive control Gö6976 (reported in Z-scores). White arrows: genes that significantly perturbed neurite initiation on both substrates, gray arrows significantly changed only on laminin, and black on CSPGs. Black dashed line separates CSPG effects between increase and decrease while gray dashed lines separates laminin effects. ***B–E***, representative images of neurons growing on CSPG (upper panels) and laminin substrates (lower panels). Neurons expressing the gene WD repeat domain 33 (WDR33) had increased neurite initiation when grown on CSPGs but decreased neurite initiation when grown on laminin (**B**), while DUS3L “dihydrouridine synthase 3-like” acted as the strongest inhibitor of neurite initiation on both substrates (**C**). SEPT8 “Septin 8” increased neurite initiation on both substrates (**D**), and dynactin 2 (DCTN2) potentiated neurite initiation on laminin but not on CSPGs (**E**). Scale bar 200 µm.

As an overview, 154 of the 832 non-redundant genes that had a significant effect on neurite initiation were arranged in a grid with vectors indicating the magnitude and direction of effects on CSPGs and laminin (Fig. 2A). The grid was constructed so that genes were ordered according to the strength of their phenotype (CSPG effects along the *y*-axis and laminin on the *x*-axis). The positive control Gö6976 was near the upper right corner (activating neurite initiation on both CSPG and laminin). 31 genes increased neurite initiation on CSPGs but not on laminin, 23 increased on laminin alone, 25 decreased on laminin and 45 decreased on CSPGs. A few genes perturbed growth on both substrates, including 15 activators and 14 inhibitors. One gene, WDR33, potentiated neurites on CSPGs and inhibited on laminin. Images of neurite growth on CSPGs are shown for neurons expressing WD repeat containing gene (WDR33, Fig 2B); dihydrouridine synthase 3-like gene (DUS3L, Fig 2C), which strongly inhibited neurite growth on both substrates; Septin 8 (SEPT8, Fig 2D), which increased growth on both substrates; and Dynactin 2 (DCTN2, Fig 2E), which potentiated neurite initiation on laminin while having little or no effect on CSPGs.

Other phenotypic changes in, for example, neurite length or the number of neurite branches were also observed. Behaviors observed for some previously identified regeneration-associated-genes are listed in Table S2. Several of these genes, including BDNF, FGFR, and c-SRC, were able to overcome inhibition after transfection. A few of the neural growth regulators displayed inhibitory phenotypes; p21, MKP3, and Jun were the strongest inhibitors.

**Figure 3:**
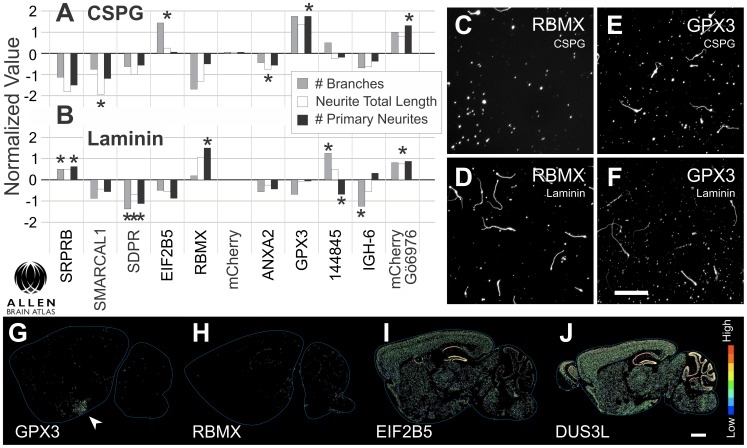
Active growth genes in cerebellar neurons and their expression pattern. ***A, B***, Phenotypic results of overexpression in cerebellar granule neurons (CGNs) confirmed by four replicates following the primary screen. Bars represent normalized values, centered on the neutral control, mCherry. Three parameters: number of branches (gray), neurite total length (white) and primary neurite count (black), are reported for transfected neurons (GFP positive). Significant results are indicated with asterisks (ANOVA). Genes had effects on CSPG (**A**), laminin (**B**), or both substrates. mCherry transfection with Gö6976, the positive control, is plotted on the far right. ***C–F***, Representative images of CGNs transfected with the gene RBMX grown on CSPG (**C**) or laminin (**D**) substrates, or the peroxidase GPX3 grown on CSPGs (E) or laminin (F). ***G–J***, Adult brain expression of four clones with significant phenotypic changes. Data were analyzed from the Allen Brain Atlas (www.brain-map.org) to determine the expression patterns and intensities of the active genes. *In-situ* hybridization demonstrated little mRNA expression for GPX3 (**G**), and RMBX (**H**), each of which promotes neurite growth. EIF2B5 (**I**), which also promoted growth had some expression throughout the brain, especially in CA1 and the dentate gyrus. Two of the inhibitory genes, SMARCAL1 (not shown) and DUS3L (**J**) had strong expression in the granule layer of the cerebellum. Expression intensity legend on the far right. Scale bar in ***C–E*** 200 µm. Scale bar in ***G–J*** 1 mm.

We selected a set of 79 genes from the primary screen to study in a secondary assay. Gene selection was based on ability to perturb the following parameters: neurite initiation, neurite length, neurite branching, neuron count, viability, and transfection rates, in either a positive or negative direction (including some of the genes listed in Figure 2A). The secondary screen challenged CGNs transfected with one of the genes on CSPGs and laminin in four experimental replicates. Sixteen of these genes had significant effects for a specific neurite outgrowth parameter (Table S3), a selection of which is depicted in Figure 3 compared to mCherry control in neurons plated on CSPGs (Fig. 3A), or laminin (Fig. 3B). The RNA binding motif protein, RBMX, was confirmed to increase neurite initiation on laminin (Fig. 3D) but not on CSPGs (Fig. 3C). On CSPGs, the extracellular peroxidase GPX3 allowed neurons to overcome inhibition, with little effect on laminin (Fig. 3E,F) both in transfected and non-transfected neurons. Since non-transfected neurons were affected, it is possible that GPX3’s action is extracellular. Interestingly, a translation factor implicated in neural development, EIF2B5 (translation initiation factor 2B5), also potentiated neurite growth on CSPGs. The genes SMARCAL1 (SWI/SNF related, matrix associated, actin-dependent regulator of chromatin), SDPR (serum deprivation response), DUS3L (dihydrouridine synthase, see table), and to a lesser extent ANXA2 (Annexin A2) and IGH-6 (LOC636126) inhibited growth on laminin and/or CSPGs. Interestingly, two of the inhibitors, EIF2B5 and DUS3L were highly expressed in the cerebellar granular layer of adult mice (Fig. 3I, J). Cerebellar enriched genes like these were expected to be present in the library at a rate of about three percent (Figure S1), opposed to the others, which were absent from the cerebellum and most of the CNS (Fig. 3G, H). The confirmed observation of their inhibitory nature suggests they may be targets to antagonize in order to enhance regeneration.

### Overexpression of GPX3 Allows Hippocampal Neurons to Overcome Inhibition by CSPGs

**Figure 4:**
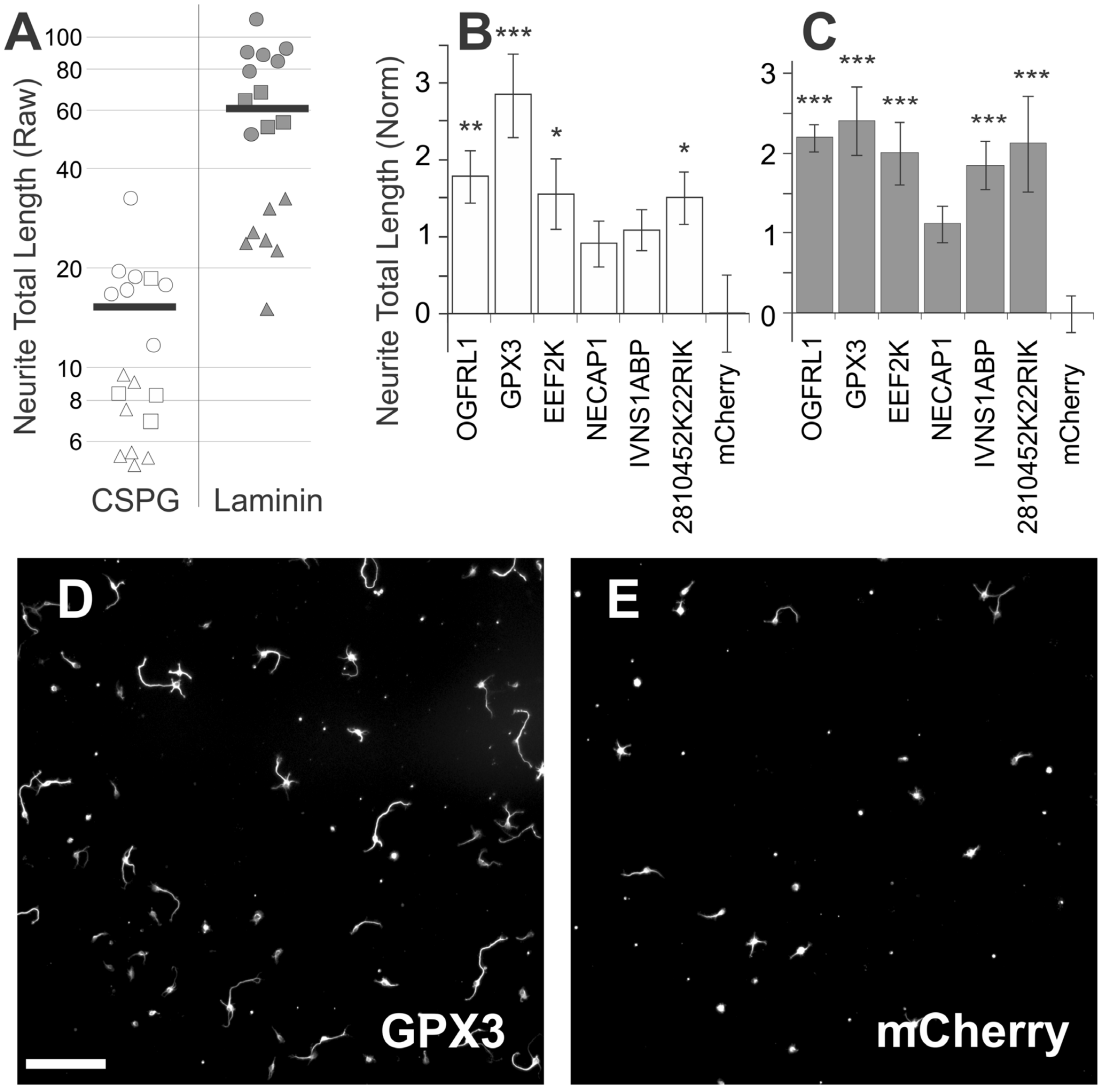
Hippocampal neurons are inhibited by CSPGs, and rescued by GPX3 overexpression. Hippocampal neurons transfected with active genes were plated on CSPG or laminin substrates. ***A***, CSPGs (white) strongly inhibited hippocampal growth compared to laminin (black) (p<0.0001, Mann Whitney U Test) in three independent experiments (triangle, square, and circle markers). Horizontal bar indicates the average neurite total length on CSPGs and laminin, 15.5 µm and 60.9 µm respectively. ***B,C*** Mean Z-Scores of transfected neurons with standard deviations, centered on the negative control, mCherry. Asterisks indicate significant effects (*, **, ***, p<0.05, 0.01, 0.001 Tukey-Kramer) compared to the mCherry control when analyzed with ANOVA (p<0.001). ***D, E***, Representative images of hippocampal neurons growing on CSPGs transfected with GPX3 (**D**) or control mCherry (**E**). Scale bar 100 µm.

To extend the results of the screen to another CNS population, primary embryonic hippocampal neurons were transfected with several genes of interest in a CSPG neurite outgrowth assay. First, CSPGs strongly inhibited hippocampal neurite growth (Fig. 4A, four fold decrease, p<0.0001 MWU). The positive control used for cerebellar neurons, Gö6976, was unable to relieve CSPG inhibition for hippocampal neurons (data not shown). Interestingly, GPX3 significantly increased growth of transfected and non-transfected neurons both on CSPG (KW p = 0.019, Dunn’s post p<0.05, Fig. 4B) and on laminin (KW p<0.022 Dunn’s post p<0.05, Fig. 4C). Neurite outgrowth was also enhanced after transfection with OGFRL1, EEF2K, IVNS1ABP, and 2810452K22RIK. GPX3 significantly increased the number of neurites, length, and frequency of initiation when overexpressed in hippocampal neurons (Fig. 4D).

**Figure 5:**
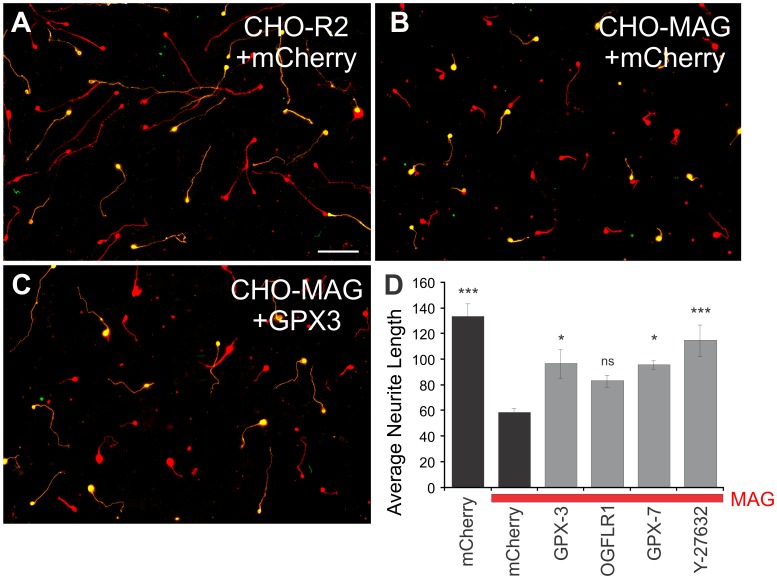
GPX3 and GPX7 significantly increase neurite length of postnatal CGNs plated on the inhibitory MAG substrate. Postnatal day 8 rat CGNs were co-transfected with the pmaxGFP plasmid (green) and the pCMVSPORT6 plasmid expressing either GPX3, GPX7, OGFLR1 or the control gene mCherry and plated onto a feeder layer of CHO cells expressing a non-inhibitory construct (R2), or the CNS myelin component, MAG. *A*. CGNs growing on CHO-R2 transfected with GFP and mCherry. *B*. CHO-MAG strongly inhibited the neurite outgrowth of CGNs transfected with GFP and mCherry. *C*. CGN neurite outgrowth is partially rescued when transfected with GPX3. *D*. Data are plotted as mean +/− SEM of 8 experiments, (One-way ANOVA, Dunnett’s post hoc, *p<0.05, ***p<0.001). Red channel marks β-tubulin positive neurons, green channel represents GFP expression, transfected neurons therefore appear yellow.

In addition to CSPGs, we showed that CGNs cultured on CHO cells expressing the myelin protein Myelin Associated Glycoprotein (MAG), had shorter neurite lengths (Fig. 5B) than CGNs growing on CHO cells expressing a control protein R2 (Fig. 5A). When GPX3 (Fig. 5C), GPX7 or OGFLR1 were transfected into the CGNs, they were able to overcome the growth inhibition of MAG (Fig. 5D), to a similar level as a positive control, the ROCK inhibitor Y-27632 (One-way ANOVA, Dunnett’s post hoc, n = 8). Thus, although the plasmids for GPX3 and OGFRL1 were identified in a screen using inhibitory CSPGs, they effectively enhanced neurite growth on another CNS inhibitory substrate, MAG.

### Bioinformatics

**Figure 6:**
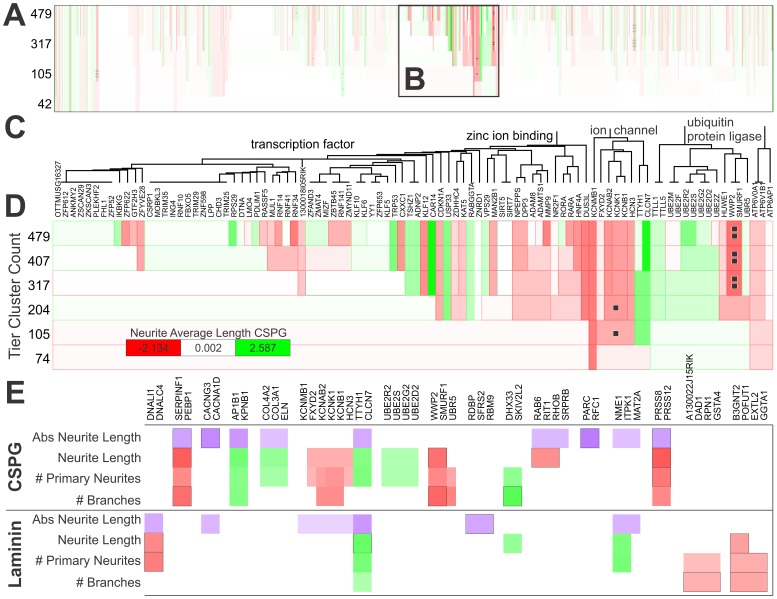
Identifying Gene Ontology Clusters that Regulate Axon Outgrowth. Tiled cluster analysis from Fig 6 run for Gene Ontology “Molecular Function” annotations on 675 genes. ***A***, Cluster heat map for the parameter neurite average length on CSPGs. 7 Tiers shown, with clusters per tier from 42 (bottom, largest clusters with most divergent genes) to 479 clusters per tier (top, smallest clusters with most closely related genes). ***B***, Region of magnification. ***C***, Dendrogram of 96 genes for region from (**B**). Four ontologies define the large classes of genes in this region (although hundreds of ontologies are present). ***D***, Cluster heat map magnified from (**B**). Individual gene clusters are defined by tiles where extent of change is color coded (white  =  control, red  =  reduction, green  =  positive). Legend in lower left corner. Single black square p<0.05, double p<0.01, (uncorrected bootstrap). ***E***, Summary table of significant gene clusters from analysis of neurite average length, branching, primary neurite count, and an absolute analysis of neurite average length (see methods). Outlines around tiles indicate higher significance.

Our primary screen resulted in quantitative functional data for a wide range of parameters. We next asked the question “Do groups of related genes, when considered together, produce significant changes in neuronal morphology?” We assume that further meaning emerges when these genes are studied as they are in reality–in a system. To artificially reconstitute the “system”, we sought to interrogate clusters using the existing functional results from the primary screen. This method [30] is the reverse of a common practice that determines representation of ontologies in a gene list compared to background [31]. We used the “molecular function” ontology information to generate a hierarchical cluster of genes. This analysis revealed that genes within some ontological clusters had directionally consistent effects on neurite outgrowth (e.g., RPS/RPL genes tended to promote axon growth; Fig. S4). Figure 6 demonstrates the results with neurite average length for neurons growing on CSPGs (Fig. 6A). A region of the ontology space (Fig. 6B), which contains transcription factors, zinc and DNA binding proteins, ion channels, and ubiquitin ligases is shown in greater detail (Fig. 6C, D). This cluster heatmap shows individual genes affecting neurite length (top tier) by color. Further down the tier, genes grouped by molecular function (i.e. transcription factor) can be seen to affect or not affect neurite length on CSPGs. Neurite length was effectively inhibited by a small group of potassium gated channels, as well as two ubiquitin ligases (Fig. 6D). It is important to note that the estimated false discovery rate for the overall screen, based on # of primary neurites, was 24% (Methods S1) and therefore this particular analysis is likely to contain some artifacts. Most of the noise was due to variations in experimental sets from different mice and different days. Higher numbers of controls on each plate would likely reduce FDR [32].

We compiled the significant results from various parameters (Fig. 6E) on CSPGs and laminin. Proteases (PRSS8, PRSS12), protease inhibitors (SERPINF1, PEBP1), and ubiquitin ligases had inhibitory effects on neurons growing on CSPGs. Two dynein genes (DNAL1, DNALC4) inhibited laminin neurite outgrowth, while the chloride channels TTYH1 and CLC7N potentiated neurite growth on both substrates.

## Discussion

Adult mammalian CNS neurons have a poor axon regeneration response after injury, while peripheral neurons in the proper context effectively elongate axons and make functional connections [33]. To determine whether specific PNS genes could improve the regenerative ability of CNS neurons, we took advantage of a sensitive technique known as subtractive hybridization to generate a cDNA library enriched in PNS genes. By combining this library with publically available microarray data, we generated a list of approximately 1300 DRG enriched genes. Many of these genes have already been identified as regeneration associated genes, including BCL2, BDNF, Cofilin, FGFR1, Galanin, Integrin alpha 3, Jak1, LIFR, LIMK, MMP9, SPP1, p21, p35, retinoic acid receptor, Socs6, Stat3 and TNFR. Rather than focus our experiments on those putative regeneration targets, however, we took an unbiased approach. Using high content screening techniques, we assayed neuronal phenotypes after a gain-of-function screen of over 1100 genes in CNS neurons on both permissive (laminin) and inhibitory (CSPG) substrates.

Many genes were observed to perturb neurite initiation, elongation, or branching, or parameters of nuclear or cell body morphology. Few of the genes with significant effects were active on both laminin and CSPG substrates. Several known growth-associated-genes were tested, and only a few were able to overcome CSPG inhibition after transfection (BDNF, FGFR, c-SRC), while other genes (p21, MKP3, Jun) displayed inhibitory phenotypes. The genes that had the strongest neurite promoting effects from the primary screen were not known as regeneration-associated genes. For example, GPX3, EIF2B5, and RBMX reliably promoted neurite growth on CSPGs (GPX3 and EIF2B5) or on laminin (RBMX). Finally, a bioinformatics analysis using hierarchical clustering of gene ontologies for the PNS enriched genes revealed potential targets for future study, including proteases/protease inhibitors, chloride channels, and ubiquitin ligases. Below we will briefly discuss some of the important points and issues raised by this study.

### Gene Identification

Many techniques can determine gene expression in specific populations of cells, including microarray, differential PCR display, 2D protein gels, serial analysis of gene expression (SAGE), subtractive hybridization of cDNA libraries, and now next generation sequencing. Microarrays can be done quickly and are reliable for clones that are expressed in high copy number. Subtractive hybridization of cDNA libraries is more laborious but allows the identification of novel genes and genes expressed at low copy number (intermediate and complex classes) [34].

### High Content Screening

High content screening (HCS) attempts to address the problems in normal high throughput drug screens in which “hits” fail after significant effort due to lack of knowledge that could have been acquired early on [35]. To obtain reliable results using HCS, large numbers of cells (typically >300) need to be analyzed for each condition. With phenotypes involving neurite lengths, branching, etc; imaging large numbers of cells presents a problem. Higher plating densities of neurons result in higher failure rates for the automated tracing algorithm, since the software (or human observer) is unable to tell which neurite emanated from which cell body. This consideration forced us to reduce plating density so that the tracing was of high quality, but the analysis occasionally suffered in power.

### Data Analysis

We asked the following question of the data, “did gene J have an effect on parameter K in some direction L, for population of neurons M, and in context N.” This relatively simple statement results in a system with 5 dimensions. Since there are over 1000 genes, 12 parameters (in data for figures and figure S1 of [30]), 2 directions, at least four populations (transfected +/−, growing neurites +/), and at least 2 contexts (CSPG/laminin), the complexity of this analysis is high. Ideally, we would have liked to separate each of these parameters, so that effects could be read out with high specificity. However, as the data were further subdivided, fewer individual cells were available for analysis. A tradeoff can be made between the dimensional depth of the analysis and the reliability gained by noise reduction when pooling by including/excluding transfected+/− or neurite+/−.

Two problems that had to be solved for data analysis were (1) transforming cell level data into treatment level data and (2) data comparison across experiments. Within one experimental replicate of a treatment, three replicate wells and ∼900 cells were present. The distribution of the values varied depending on the parameter. For example, soma area was the most normal, nuclear intensity was bimodal, neurite count was discrete exponential, and neurite length was the most complicated (similar to exponential distribution). When the cell numbers were high, the mean of the pooled values for all the cells in three wells was repeatable. Other approaches, such as Kolmogorov Smirnov tests [36] were attempted, but were somewhat noisier than the mean. The second issue, comparison of data from one experiment to the next, was addressed using normalization. Z-scores are centered (z = 0) on the control population’s mean and its units are that of the control population’s standard deviation. The control population could in principle either be a negative control treatment, or some subset of the entire population. Because we could not establish, a priori, a treatment that was neutral with respect to multiple parameters, we pooled all of the experimental genes on a plate as the control population for normalization. This resulted in a symmetric Z-score distribution with a mode near 0.

### Gain vs. Loss of Function

In this study, we used overexpression to characterize the effects of specific gene products on neurite outgrowth. However, it is equally likely that neurite outgrowth could be enhanced by the reduction of specific mRNA populations. RNA interference (RNAi) is a common strategy often used to knock down particular genes. The main advantage of the RNAi technique is that a gene loss is likely to access the endogenous function of that gene. However, this also has several disadvantages: knockdown is variable at the protein level, genes often function in redundant groups, essential genes are difficult to knockdown, and RNAi has substantial off-target effects [37].

Gain of function, on the other hand, has several advantages. It is unlikely to kill cells since it is limited by endogenous regulatory mechanisms. Redundancies leading to a silent knock-down phenotype are overcome by overexpression. One potential disadvantage is that unnatural phenotypes might result. For example, overexpression of enzymes (such as kinases) could increase binding of improper substrates, or certain isoforms might act as dominant negatives when overexpressed. As with loss of function, gain of function is sensitive to negative feedback triggered by expression changes and pathway crosstalk.

### Known Neuronal Growth Regulators

Regeneration associated genes have been identified by many groups. We compiled a list of genes from the literature pertaining to neurite growth. Thirty-seven of the known regulators of neurite growth were identified by us as DRG-enriched genes and assayed for neurite growth on laminin and CSPGs (Table S2). A few of these genes are worth noting. p21, the CDK regulatory gene (CDKN2A) has been previously reported to be downstream of NGF in PC12 cell differentiation [38]. In our study, overexpression of p21 in neurons strongly inhibited their outgrowth on both laminin and CSPGs. BDNF, when overexpressed in cerebellar neurons, enhanced growth, branching and primary neurites for neurons growing on CSPGs. BDNF is classically an extracellular signaling molecule, although its expression in developing neurons has led to speculation about intracellular mechanisms of action [39]. The dual specificity phosphatase MKP3 (DUSP6) has been implicated in neuritogenesis in PC12 cells [40], and was inhibitory on both substrates when overexpressed in neurons. It is not clear why DUSP6 (and others) were strong inhibitors in our assay, but it may be due to the lack of activators or other signaling pathway members in the cerebellar neurons.

The transcription factor c-Jun also mediated inhibition on both substrates, and has been indicated in both cell death and regeneration [41], [42]. Although the PDGF receptor beta subunit (PDGFRB) was observed to potentiate neurite growth on CSPGs in the primary screen, that effect was not significant on CSPG in the secondary screen. RelA and c-Src [43], [44] were both observed to potentiate growth in our screen.

### Novel Neuronal Growth Regulators

After extensive primary and secondary screens, several novel genes were identified based on their abilities to modify neurite outgrowth on inhibitory and/or permissive substrates. Examples are discussed below.

#### GPX3

Glutathione Peroxidase 3 (GPX3) is part of a family of selenium containing antioxidant enzymes that work to maintain the oxidative homeostasis and protect the cell from oxidative stress. The GPX family can convert hydrogen peroxide to water and neutralize lipid peroxides, thereby preventing them from forming aggressive free radicals. Consistent with this, cellular glutathione peroxidase, GPX1 has been implicated as having a critical neuroprotective role in many brain disease and injury models, including Parkinson’s disease, dementia [45] and ischemia [46].

GPX3 is known as plasma glutathione peroxidase and has not been investigated in the context of brain disease and injury. One hypothesis for GPX3’s action is a neuroprotective effect, consistent with its effect on both transfected and un-transfected neurons. Considering GPX3’s similar effects in both cerebellar and hippocampal neurons on CPSGs, it is likely that its effects are linked with overcoming inhibition. It is plausible that GPX3 is sequestering sources of free radicals in the extracellular space, leading to a global increase in cell viability in the entire culture well. This effect would also be desirable in a spinal cord injury model: by decreasing the oxidative stress of the neurons in a combination paradigm, neurons may be more responsive to regenerative therapeutics. It is becoming clear that the oxidation state of proteins may be involved in signaling, and not just cellular stress [47]. The mechanism of GPX3 action still needs to be elucidated.

#### RBMX

RBMX is an RNA binding motif protein on the X-chromosome. It is involved in pre mRNA splicing, transcriptional regulation [48], and in particular plays a role in xenopus and zebrafish neural development. A screen in *Xenopus* identified many important RNA binding proteins, and after knocking down RBMX, the authors determined that it is necessary for anterior neural plate patterning, neurogenesis, and neural crest development [49]. Knockdown of RBMX in zebrafish led to small heads, defective eyes, and loss of specific markers for the fore- and hindbrain [50]. RBMX may regulate expression of growth associated proteins specific to peripheral gene development (neural crest), which are normally not well expressed in CNS neurons.

#### EIF2B5

EIF2B5 is one of 5 subunits of EIF2B, a guanine nucleotide exchange factor (GEF) that activates eukaryotic translation initiation factor 2 (EIF2). In PC12 cells, induction of neural differentiation with NGF or EGF activates protein synthesis and leads to phosphorylation of EIF2B by GSK3B and casein kinase II [51]. Thus, EIF2B could be a new downstream target of GSK3B that is important for process outgrowth. In our screen, EIF2B5 overexpression increased neurite length and branching, especially on CSPGs. Upregulation of protein translation is likely to be critical for effective axonal regeneration [52].

#### DUS3L

This gene encodes a relative of the dihydrouridine synthases, which catalyze the formation of dihydrouridine, a modified base found in the D-loops of many tRNAs. DUS3L contains two zinc finger domains [53], but its function is currently unknown. It was one of the strongest neurite growth inhibitors in our screen. Interestingly, it has very high expression in the brain, and specifically in the granule cell layer of the cerebellum (Fig. 3J). This gene was then probably mistakenly picked as differentially expressed. Further experiments are required to understand its mechanism of action.

### Gene Families

We used novel techniques in bioinformatics to determine if families of particular genes had significant effects based on the activities of the family members. This technique was designed to identify particular genes and gene classes as targets of future investigation. Gene ontology classes were used to link related genes, since little pathway information was available for them.

Two serine proteases (PRSS8, 12) as well as two serine protease inhibitors (SERPINF1, PEBP1) were found to further inhibit growth on CSPG substrates. The protease inhibitor PEDF (SERPINF1), has been identified as a neurotrophic factor, and seems to be involved in apoptosis inhibition of mouse cerebellar neurons. Interestingly, it protects young, but not old cerebellar neurons [54]. It is yet to be explored whether peripheral and central neurons might also be differentially protected. One of the proteases, PRSS12, known as neurotrypsin, is preferentially expressed in motor neurons, upregulated during recovery from facial nerve axotomy [55], and is involved in synaptic plasticity and dendritic remodeling [56], [57]. Its close relative PRSS8 was also inhibitory in our assay.

As mentioned above, the initiation factor EIF2B5 was confirmed to overcome inhibition when overexpressed in cerebellar neurons. The elongation factor EEF2K and several of the ribosomal subunits (RPS15, RPS19, RPL41, and RPL10) also potentiated growth on CSPGs. Evidence from Christine Holt’s group supports local axonal translation [58] As axons traverse tissue to find their ultimate target, various cues change the direction and the expression of surface receptors on the growth cone, requiring instantaneous changes in translation [59]. Specifically, we now know that guidance machinery like the netrin receptor (DCC) is part of a protein translation complex that can be controlled through receptor ligation [60]. Others have shown the importance of translation in terms of mTOR [52]. EIF5A (not screened), has been shown to be involved in neuronal growth and survival in brain cultures [61], and yet another, EIF4E, was identified as being phosphorylated downstream of Ras during PC12 cell differentiation [62]. The required regulation of translation could be deficient in injured central neurons.

Protein degradation is a critical way in which neurons regulate the behaviors of their axons and dendrites. The ubiquitin protease system uses a set of enzymes to identify, tag and degrade specific proteins through the proteasome. E2 ubiquitin conjugating enzymes, including UBE2G2, UBE2D2, UBE2R2, UBE2S were identified as a group for allowing CGNs to increase the number of primary neurites and average length when grown on CSPGs (Figure 6E). These enzymes carry ubiquitin, but aren’t considered to be target specific. This finding implicates the ubiquitin proteasome system or autophagy in the sensitivity of neurons to CSPGs. E3 ubiquitin ligases are of particular interest, since they confer substrate specificity by bringing the substrate and the E2 within range. In this screen WWP2, SMURF1, and UBR5 all had negative impact on neurite parameters on CSPGs. WWP2 is a Nedd4-like E3 ligase and is particularly interesting considering the recent findings that Nedd4 regulates PTEN, which in turn directly regulates PI3K and axon branching [63]. Nedd4 has also been shown to target neuronal elongation through Rap2a [64]. WWP2 may function similar to Nedd4 in CGNs. SMURF1 (SMAD specific E3 ubiquitin protein ligase 1) was previously identified to have a direct role in neurite elongation through its degradation of RhoA [65]. A third PNS E3 ligase in the group, UBR5 (ubiquitin protein ligase E3 component n-recognin 5), has yet to be studied in neurons.

In other families we identified just two members as having a significant effect. These included two chloride channels (TTYH1, CLCN7), which increased growth on CSPG and on laminin, and two subunits of dynein (DNALI1, DNALC4), which decreased growth on CSPG. Dynein mutations have been shown to result in severe degeneration of motor neurons [66], and the effect seen here may be due to a similar cargo/transport deficit caused by overexpression of just one of the subunits at a time.

### Conclusion

The regenerative ability of CNS neurons may be improved by forcing expression of genes normally expressed by PNS neurons. Ours is the initial study to combine the powerful techniques of subtractive hybridization and high content screening in primary neurons to test this idea. CNS neurons were directly manipulated with exogenous PNS gene expression, and assayed for their ability to send out processes on CSPGs – the most potent known inhibitory substrate associated with CNS injury. Known regeneration associated genes (BDNF, p21, Jun, RelA, c-Src) modified the neurite growth of CNS neurons after their overexpression. Novel genes GPX3, and EIF2B5 were confirmed to relieve the inhibition of neurite growth on CSPGs for cerebellar neurons. Importantly, GPX family members also relieved inhibition on a well-established myelin inhibitor (MAG), as well as CSPG inhibition in hippocampal neurons. By clustering genes using GO terms we found that several gene families, such as regulators of transcription and ubiquitin pathways, may underlie key intrinsic differences between PNS and CNS neurons that account for their different regenerative potentials.

## Methods

All procedures using animals were approved by the University of Miami Animal Care and Use Committee.

### Molecular Biology

#### cDNA library construction

The cDNA library construction and preparation was detailed elsewhere (Smith et al., 2011). Briefly, C57/Bl6j postnatal mice were anesthetized and decapitated. DRG neurons were dissociated (see below), and whole cerebella were gathered. DRG were collected from culture after 48 hours, and then homogenized in Trizol reagent (Invitrogen, Carlsbad, California, 15596). Cerebella were placed directly in Trizol and homogenized. Total RNA was extracted, and proteinase K (Roche, Mannheim Germany, 03-115) was added to remove all RNase. Poly-(A)+ mRNA was purified from the total RNA by Oligotex mRNA kit (Qiagen) with two rounds of selection. Invitrogen’s SuperScript Choice System for cDNA synthesis was followed by poly-T primers, and clones were blunt-end ligated into a phage vector. DRG cDNA was separated into three size fractions prior to vector ligation by gel and AgarACE digestion (Promega). Lambda vector was Lambda Zap-CMV XR (Stratagene). Packaging was done with Gigapack III Gold Packaging extracts (Stratagene), then titered, in vivo amplified, and stored. Mass excision of the resident phagemid resulted in a pCMV-Script EX mammalian-expression plasmid library.

#### Subtraction library

Details of the subtraction and sequencing are described in [22], and are based on [34]. Purified DRG library plasmid was converted to single stranded plasmid by Gene II enzyme (Invitrogen 10356-020) and Exo III. Cerebellum (driver) library was amplified by PCR using T3 and T7 primers. 100 ng of DRG tester single stranded circles were hybridized with 2.5 µg PCR product at 30°C for 88 hours. Blocking oligos were used to keep common sequences from hybridizing. The mixture was then run through a Hydroxyapetite (HAP) column which bound to partially double stranded, but not the un-hybridized single stranded DNA. Recovered single stranded plasmids were desalted and concentrated. PCR was used to extend the T3 primer, generating partially double stranded plasmid, which was transfected into DH10B electro-competent bacteria. To reduce the redundancy of the subtraction library, the first ∼800 sequenced clones were used to generate a new driver pool, and were serially subtracted from the starting subtraction library, allowing the less abundant clones (the complex class) to be sequenced.

#### Sequencing

Glycerol stocks in 1.5 tubes from the subtraction library were stabbed and spread across 100 mm Petri dishes with LB agar containing 50 µg/ml kanamycin, then grown overnight at 37°C. Individual clones were picked and inoculated into one well of a 96-well deep well block (Qiagen), which was prefilled with 1.4 ml 2xyT. Plates were grown in 37°C, shaking at 310 RPM for 14–20 hours. Glycerol stocks were derived from 50 µl of the bacterial culture, combined with 50 µl of 2xYT media and 20% glycerol. Glycerol stock plates were grown in a 37°C incubator for 1 hour, and then stored in a −80°C freezer. Plasmid DNA was prepared from the bacterial cultures using a Qiagen BioRobot (see cDNA Plasmid Purification) and resuspended in warm elution buffer. Four of the 96 clones were quantified with a cuvette spectrophotometer (Eppendorf), which showed an average 300 ng/µl concentration. Applied Biosystem’s BigDye Terminator (v3.1, 4337455) kit was used for sequencing, according to the manufacturer’s instructions. Briefly, sequencing master mix was made, for each reaction, consisting of: 8 µl of 12.5% glycerol, 4 µl 5x sequencing buffer, 1.5 µl of 1 µM T3 Primer (5′ end vector), and 1 µl of Big Dye component. 150 ng of template was added in 5.5 µl water. Reactions were made up in semi skirt 96-well PCR plates (GeneMate), sealed and placed in an MJ Research PTC-200 thermo-cycler with the program: 1 = 96°C 5 m 2 = 96°C 15s 3 = 53°C 5s 4 = 60°C 4 m 5 =  Goto 2, 34 times 6 =  Hold at 4–10°. After the sequencing reactions were completed, we precipitated them with a buffer consisting of 35 ml absolute ethanol, 15 ml molecular quality water, and 10 µl of 1 M MgSO_4_. 75 µl of the precipitation buffer was added to each well of the sequencing reaction in the PCR plate. We placed the plates in the dark at room temperature for 15 minutes, followed by thorough vortexing and centrifugation at 2600 x g for 15 minutes at 4°C. Next we carefully folded paper towels, 2 cm thick, and placed them on top of the un-sealed, un-covered PCR plate. Plates were inverted onto the paper towels to pour out all of the liquid, and placed upside down in the centrifuge. The plates were then spun at 200 x g for 1 minute at 4°C. The PCR plates were then sealed and shipped to the W.M. Keck Center at the University of Illinois for sequencing.

Raw sequence data and Phred scores were uploaded from the facility, and analyzed by EST Express (Smith et al., 2011), which returned UniGene and Entrez Gene identifiers for each genes on the plate. The sequencing of 2,016 clones had a failure rate of 6% bad sequence reads, and 10% vector only clones.

#### Microarray

Microarray technique and analysis is described in detail elsewhere [22]. Briefly, data from laser capture micro-dissected DRG neurons was provided by Peeters [67]. Cerebellar DNA was obtained from three P11 mice. Affymetrix Murine Genome U74AV2 chips were used and samples were normalized using the Robust Multi-Array average by RMA Express (http://rmaexpress.bmbolstad.com, [68]). Normalized intensities were pooled in Spotfire DecisionSite (TIBCO Software Inc., Palo Alto CA) and mean DRG/Cerebellum ratios were calculated.

#### Q-PCR validation

To validate the reduction of cerebellar genes by the subtraction, quantitative PCR (Q-PCR, reverse transcription followed by real-time PCR) was used. The test population was 6 genes sequenced more than once, 12 random genes picked from the subtraction library that were called absent in both samples by microarray, and 8 genes of interest. DRG neurons were prepared as below (DRG Preparation), images were taken to confirm viability of the neurons. Whole cerebella were extracted from P8 mice and homogenized. Trizol reagent was used to isolate total RNA from the DRG neurons and whole cerebella. 100 µl of 60 ng/µl RNA was purified with 260/280 = 2.1 and 260/230 = 0.89 (by Nanodrop spectrophotometer, Thermo Fisher) after RNeasy (with superasin) kit. Reverse transcription was done with Invitrogen Superscript kit (18064-022) using random primers. PCR primers were designed using primer-3 (http://frodo.wi.mit.edu/), and several were ordered for each test gene. Primers were tested in a gradient cycle, and the optimal annealing temperature was determined. 38 cycles of Q-PCR were run on an Eppendorf Mastercycler ep realplex (Hamburg, Germany), using Epicentre master mix (TAQurate GREEN Real-Time PCR MasterMix, TM046400).

#### Allen brain atlas validation

Allen brain atlas [24], [69] has catalogued the expression of genes, genome-wide for the entire adult mouse brain. In addition, they have established a “neuroinformatics” pipeline [70] to carefully analyze and store the data. Their in-situ hybridization was cross-validated to both BGEM (http://www.stjudebgem.org/web/mainPage/mainPage.php) and other data [71]. The images shown in Figure 3 are “expression-mask” images from sagittal sections of the antisense ribo-probe, taken ∼2.5 mm lateral of the midline. For the supplemental figure, average density readings extracted for each brain region were compared from cerebellum to the other brain regions to produce a ratio. For the genes in the screened library, expression ratio comparing most other brain tissue to the cerebellum showed a cerebellar reduction (consistent with the design of the library). To ensure that the expression map wasn’t biased in this direction to begin with, a random set of 1000 genes was used as background and ANOVA with Dunn post-tests were performed.

#### Full length plasmid cDNA library

A cDNA library in glycerol stock from the NIH Mammalian Genome Collection [23] in 96-well format was purchased from Open Biosystems (ThermoFisher. Huntsville, Alabama) with >16,000 clones from human (IRAT) and mouse (IRAV). The library was replicated and both the original and daughter plates were stored in −80 degree freezers, as previously described in (Buchser et al., 2010). A custom program written in Excel VBA took gene lists and generated a protocol for the Qiagen BioRobot-3000 (Germantown, Maryland). This instrument then picked clones from the glycerol stock plates into the “screened library”, which totaled 16 x 96-well plates. During the cherry-picking process, a set of 10–12 plates were thawed, wiped, un-capped, and the foil cover carefully removed. Disposable tips were used to automatically inoculate cultures in 96-well blocks. During the process, glycerol stocks for mCherry [72] were also picked and inoculated into specified control wells on the plate. Other wells were left empty, to facilitate non-transfected controls and to allow other clones to be tested. In addition, the empty wells provided a visual confirmation that no cross-contamination occurred during the process.

#### Plasmid preparation

QIAprep 96 Turbo BioRobot Kit (Qiagen 962141) was used to produce transfection quality plasmid. Briefly, two deep well blocks from the kit were filled with 1.4 ml Terrific Broth (Invitrogen 22711) and 150 µg/ml ampicillin (Invitrogen 11593) in each well. Thawed glycerol stock plates were inoculated into fresh media with a 96-pin replicator tool (Nalge Nunc 250520. Rochester, NY). Plates were incubated for 20–24 hours 37°C, shaking at 300 RPM. The plates were spun down serially such that the pellets were overlaid and concentrated. The pellets were resuspended in P1, and the manufacturer’s instructions were followed. Elution was performed at room temperature, with 110 µl of endotoxin free water.

Plasmid was generally purified at ∼300 ng/µl with an average 260/280 ratio of 1.8. Plasmid concentration was analyzed with NanoDrop spectrophotometer (Thermo Fisher. Wilmington, Delaware). Agarose gels were run on several complete plates, revealing expected bands and little degradation (data not shown). Before transfection, the concentration was standardized across a plasmid plate by adding endotoxin free water to bring all clones with high concentrations down to 300 ng/µl. Clones with concentrations below 225 ng/µl were not included in the analysis.

### Cell Culture

#### Cerebellar culture

Homogenous postnatal day 8–10 C57/Bl6j mouse cerebellar granule cell cultures were prepared as previously described [22]. Briefly, cerebella were harvested and minced with a new razor blade. The dissociation buffer used in all steps was room temperature Hibernate media (BrainBits, HE-Ca 500. Springfield, Illinois). The cerebellar pieces were incubated in 0.05% Trypsin-EDTA (Invitrogen 25300) for 15 minutes in a 37°C water bath, without swirling. The trypsin was inactivated by adding horse serum to 10% and diluted with Hibernate. The cells were triturated sequentially with large and small-bore flame-polished glass pipettes in the presence of 0.5 mg/ml DNase I (Sigma D5025). The cells were spun and resuspended in Hibernate for counting. Centrifugation steps were room temperature 80 x g for 7 minutes. Solutions and cells were kept at room temperature throughout the procedure. Preparations yield >90% cerebellar granule neurons.

#### DRG preparation

Postnatal C57/Bl6j mice were euthanized, decapitated, and placed on ice. The cerebellum and/or brain was dissected and placed in cold hibernate for other cultures. Vertebral columns were removed and cleaned in L15 media (Invitrogen, 11415) in a dish resting on ice. The vertebral column was bisected medially with a fresh razor blade, and the halves were stored in fresh Hibernate. The hemi-spinal cord was removed, and individual DRGs were pulled from the intervertebral foramen using forceps and placed into a small dish with 2.4 U/ml Dispase, 1,000 U/ml Collagenase, and 0.05% Trypsin-EDTA (Invitrogen 25300). DRGs were incubated at 37°C for 45 minutes, and lightly swirled every 15 minutes until enzyme inhibition by fetal bovine serum (FBS Invitrogen 16000). DRGs were triturated in the presence of 0.5 mg/ml DNase I (Sigma D5025), rinsed with L15 or hibernate media, resuspended in a small volume of L15 media, and counted.

#### Hippocampal culture

Embryonic hippocampal cultures were prepared as described previously [30]. Briefly, adult mothers, pregnant with E18 Sprague-Daley rats, were euthanized and the embryos were dissected in fresh Hibernate media supplemented with B27 (Invitrogen 11602). Isolated Hippocampii were transferred to Hibernate media without B27 and incubated for 15 min at 37°C with 0.05% Trypsin (Invitrogen 25300), in the presence of DNase I at final concentration of 0.5 mg/ml (Sigma D5025). The tissue was then washed 5 times with the Hibernate media supplemented with B27 and triturated until no clumps were visible (about 5–10 times). Dissociated neurons were counted and used for transfection during the next 2 hours.

#### CHO-MAG

The CHO cell lines were established by stable transfection of CHO cells with the pSHL plasmid containing the large (L)-MAG isoform DNA in either the 5′–3′ (CHO-MAG) or the 3′–5′ (CHO-R2) orientation followed by gene amplification using the dihydrofolate reductase/methotrexate strategy. The CHO-MAG cells therefore express the L-MAG isoform on their cell surface, while the CHO-R2 cells are the control cells and express the reverse peptide sequence of L-MAG on their surface. The CHO cells were a gift from Marie Filbin [73].

### Transfections

#### High throughput transfection

Clones of the screened library were purified from bacterial cultures in 96-well format (see above). Transfection of plasmid was performed with an average concentration of 272 ng/µl, and 260/280 ratio ∼1.8. Cerebellar transfection technique was described previously [74]. A Harvard apparatus/BTX electroporation plate (2 mm gap) was sprayed with alcohol and left to dry in a laminar flow hood. 15 µl INB was added to each well, then 15 µl with 4.5 µg of DNA was mixed in for each electroporation well. 300,000 to 315,000 dissociated cerebellar neurons (on ice for up to four hours post preparation) were resuspended in 35 µl INB per well, and distributed on top of the INB/DNA for a total of 65 µl per well. GFP reporter (Lonza Amaxa, pMax GFP) was added at 0.9 µg per well (to all wells except the transfection control wells). The electroporation plate was sealed with 3 M tape pads, and tapped several times on each side to ensure mixing. Plates were electroporated with 1 pulse at 350 Volts, 850 µs. After transfection, 80 µl of room temperature Hibernate was immediately added to each well as recovery solution. These steps were performed very quickly, averaging ∼4 minutes total for 1 96-well plate. One electroporation plate contained 12 controls and 84 test clones. Transfection efficiencies averaged 10.4% (95CI 10.2–10.7%) for neurons plated on laminin, and 14.3% (95CI 14.0–14.7%) for neurons on CSPGs across treatments.

#### Hippocampal transfection

Transfection of embryonic hippocampal neurons was accomplished by Amaxa 96-well Shuttle nucleoporation system (Lonza, Walkserville, Maryland) following the manufacturer’s instructions [30]. Briefly, the Amaxa 96-well nucleoporation plate was loaded with the mixture of 75,000 neurons in 20 µL of Amaxa transfection solution, and 600 ng of experimental DNA (including 150 ng GFP reporter with) in a volume of 2.1 µl. The rat neuron transfection, “high efficiency” program was used, and the neurons were recovered with 80 µl of ENB + HEPES (20 mM Invitrogen 15630). In the CHO-MAG experiments, the CGNs (dissociated from P7-9 Long Evans rat pups) were suspended in INB and transfected with 4 µg of the pCMV-SPORT6 plasmid and 1 µg pmaxGFP plasmid. The pulse parameters were a single pulse at 300 V, 1 ms in length using the Harvard Apparatus/BTX (as above).

#### Plating

Neurons were grown in 96-well plates (Perkin Elmer, 6005182. Waltham, Massachusetts) coated with a base substrate of 10 µg/ml Poly-D lysine (Sigma P7886) overnight, and rinsed 5 times with water. Laminin plates were produced with 10 µg/ml laminin (Sigma L2020) diluted in HBSS (Invitrogen 14175) overnight, rinsed 5x with HBSS. CSPG plates were a mixture of 10 µg/ml laminin and 1 µM mixture of CSPG [75] in HBSS overnight, rinsed 5x using slow pipetting speed (Rainin). Where indicated, Gö6976 (Calbiochem, EMD Biosciences, Darmstadt, Germany, 365250) was added at 0.5 µM, diluted directly in the media. 36,000 CGN cells were added to each well. Plating media was made in DMEM (Invitrogen, 11965), 10% FBS (Invitrogen 16000), 25 mM HEPES (Invitrogen 15630), 12.5 mM KCl glutamate, penicillin/streptomycin, and sodium pyruvate. The same lot of FBS (307932) was used throughout the screen. The number of neurons was decreased on CSPGs, suggesting poor adhesion to the substrate. For hippocampal neurons, 7,500 cells (live) were added to each well. Enriched neurobasal (ENB), modified from [76] included Neurobasal (Invitrogen 12348), penicillin/streptomycin, insulin (Sigma I6634 5 mg/ml), sodium pyruvate (1 mM), transferrin (Sigma T1147 100 mg/ml), BSA (Sigma A4161 100 mg/ml), progesterone (Sigma P8783 60 ng/ml), putrescine (Sigma P7505 16 mg/ml), sodium selenite (Sigma S5261 40 ng/ml), triiodo-thyronine (Sigma T6397, 1x), L-glutamine (1 mM), N-acetyl cysteine (Sigma A8199NAC, 5 mg/ml), and B27 (Invitrogen 11602).

#### Fix/Stain

Neurons were incubated in 37°C, 5% CO_2_ incubators for 48 hours. Plates were removed and immediately fixed with room temperature fixative (4% paraformaldehyde, 4% sucrose in PBS) by removing 80 µl and overlaying 120 µl of fixative at the slowest speed. Plates were incubated 4 hours in 4°C, then rinsed with PBS and stained for E7 beta-tubulin (produced in-house) and Hoechst dye (Invitrogen 33342). Rinsing was performed with a Biotek platewasher (BioTek, Winooski, VT. ELx405).

#### Imaging/Tracing

Cellomics Arrayscan VTI (Thermo Fisher Cellomics; Pittsburgh, Pa) was used to automatically image 9 fields in 48 wells of the assay plates at 5x magnification and 1024x1024 resolution in three different channels: nuclear staining (Hoechst), neurite staining (tubulin), and the reporter gene (GFP). Images were traced automatically using the Neuronal Profiling Bio-application version 3.5. Many parameters were reported by the tracing algorithm, including nucleus (Hoechst intensity, nuclear area), cell body (tubulin intensity, cell body area, cell body shape), neuronal parameters (number of primary neurites, length of all the neurites “total neurite length”, length of the longest neurite, number of branches), and population parameters (percent of neurons which initiate neurite growth “neurite initiation”, percent of transfected neurons, ratio of tubulin positive neurons per Hoechst positive nucleus).

### Data Analysis

#### Data aggregation/Storage

Raw data was managed by the Cellomics Store, which consists of an SQL database and a network-attached fileserver (HP). Raw data consisted of metadata associated with scanning and tracing (exposures, focus offsets, thresholds), raw images and the results of the tracing. Additionally, cell and well level results were exported in tab delimited text and Spotfire formats and stored on a separate network-attached fileserver, organized by experiment with accompanying Excel tables listing how each well was treated.

Spotfire was used to associate the treatment variables (the mapping of plasmid transfection to each well) and perform basic quality control, including checks for tracing errors, low and high-density wells, cell clumps, and plating errors.

Over 3,600 transfections were performed, as part of 38 experiments. The results were validated, and whole experiments were rejected that didn’t meet quality control standards (usually due to poor growth on laminin-coated plates). Images were hand curated to ensure tracing accuracy. First, wells that contained outliers in any parameter were examined for tracing errors. Next, ∼10% of all the wells, randomly chosen, were examined. Most of the wells identified with poor tracing suffered from bad segmentation, which was easily corrected by re-analysis with new thresholds. In all, about 2% of the wells were excluded because of tracing errors that were not easily corrected (high cell density, too low signal, artifacts in the well).

#### Transfection

Transfection was monitored by the co-expression of a reporter gene, GFP, added to all wells except the transfection control well, at a ratio of ∼1∶5 with the gene of interest. In this condition, an estimated 75% of neurons that were expressing GFP were also expressing the gene of interest. We verified that the transfection effectively produced protein for several control genes, including fluorescent genes GFP in pMax vector, Venus in pcDNA3, and mCherry in pcDNA3 and pSport6. L1CAM in pCDNA3 was also tested, as well as GFAP, NCAM, MBP, and Vimentin in pSport (data not shown).

Each electroporation plate had a set of controls including a transfected well with no cDNA added to establish background green intensity and transfection thresholds. The green intensity of no-cDNA wells was fit to the tubulin intensity, which usually bled through from the red channel. The fit was used to adjust the green intensity, effectively removing any red contribution, and then a threshold was set at 2 standard deviations above the log of the adjusted green intensity in the control.
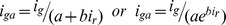
i_ga_  =  intensity for green channel, adjusted; i_g_  =  intensity for green channel; i_r_  =  intensity for red channel; a and b are the coefficients of the curve fit (for linear or exponential fits).

#### Analysis

Data was structured in terms of an “experiment” which we defined as a unit distinct from other experiments due to unique animals, transfection, and date. An experiment was done on one 96-well electroporation plate, with ∼80 experimental cDNAs and controls. This plate was split into twelve assay plates, 6 for laminin and 6 for CSPG substrates. Only wells B3 through G10 were utilized for the cells, (but media was added to all the wells) to reduce edge effects. An individual electroporation well was split into three replicate wells on each substrate. At least two *experimental* replicates were performed, but some of the genes lost the second replicate (or even both) due to quality control.

#### Normalization

Cells were pooled across replicate wells, and the average of the pool was determined. The average and standard deviation of the population of treatments for all of the transfections within one experiment (excluding no cDNA and other controls) was used as the normalization control, in the equation below. Normalization was performed separately for laminin and CSPG substrates.
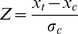
x*_t_* is a treatment’s mean, x*_c_* is the control mean (in this case the control is the entire set of experimental neurons within one experiment). 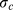 is the standard deviation of that same control population. Normalization transformed the treatment level data into z-scores. In experiments using hippocampal neurons, the Z scores for each treatment were subtracted from the Z score of mCherry, re-centering the data on the negative control (rather than the population mean). This procedure was used because the genes tested on hippocampal neurons were all predicted to alter neurite growth, based on the primary screen. Figure S5 shows that the normalized variables (relative) performed better than the raw (absolute) ones.

Many different parameters were measured; these are listed in the “data for figures”. Using strictly standardized mean difference (SSMD), we determined the optimal parameters to report (Figure S5, Table S4), including neurite total length and %neurons with neurites. SSMD allowed us to determine which variables produced the strongest differences between the negative and positive controls. Principal component analysis (PCA) was also run to determine which parameters were related and which formed distinct measurements. The PCA confirmed that most of the neurite parameters are highly related and did not produce a new component with better SSMD in comparing the data.

#### Statistics

Experiments were done to determine which type of statistical analysis was most applicable. On a cell-by-cell basis within one experiment, many treatments were significant (for example, in t-tests of each treatment compared to pSport mCherry, allowing for all cells to be considered, 84% of the treatments had p<0.001). The high positive rate and the non-normal distribution of the data, the high cell number, and the high variability of neuronal morphology from neuron to neuron made this method unacceptable. Instead, the much more conservative Mann Whitney U Test was applied to normalized treatment level data. Three genes with several parameters on both substrates were chosen that had low variability and normalized values near 0: UBE2V1, SLC25A3, CDS2. Mann Whitney was used against these “center” genes to determine significance of each of the clones. For other analyses, as indicated, Mann Whitney was compared to pSport mCherry control. Statistics were carried out in Spotfire DecisionSite.

### Bioinformatics

#### Vector grid

The vector grid is a scatter plot that uses arrows (vectors) as markers, as in Figure 2. The markers are placed into the grid based on a pixelization technique, whose concept is from [77]. Any two continuous or discrete parameters can be used for the two axes. A custom algorithm (Visual Studio 2008, C#), matched the standard Cartesian plot of the two axes to a grid, to minimized the “distance” from the original position to the new grid-mapped position. If there are 9 data points, a grid of 3×3 is constructed, and those bins are filled with the 9 data points such that their ordering is similar to the original x/y plot.

#### Performance of known regeneration associated genes

Regeneration associated genes were listed in Table S2. Each gene was compared (per substrate) to the “center genes” (UBE2V1, SLC25A3, CDS2) by Mann Whitney U test. Any gene that was significant (and Z had the same sign) for either all cells, only GFP+ cells, or only Neurite+ cells, was labeled. ++ is p<0.05 (MWU compared to centers), + is p<0.1. The weaker alpha of 0.1 was used to indicate the direction of the effect, even if not significant.

#### Gene ontology cluster analysis

Gene ontology (GO) terms http://amigo.geneontology.org/were acquired in Feb 2009 through Entrez gene. Each GO term was mapped into a hierarchy based on its “is_a” definition. For example, the GO term “Protein Tyrosine Kinase Activity” is a child of “Protein Kinase Activity” which is a child of “Kinase Activity”. A table was constructed which listed genes in each row and each unique GO term in columns. Then if the gene possessed a GO term, or possessed a child of the GO term listed in the column, it was given a 1. An example of this table is shown in Fig. 6. The table was imported into Spotfire and hierarchical clustering (using correlation and UPGMA) was performed. A custom C# program was developed that sliced through the hierarchy at various distances (termed tiers). The program operates on one unit at a time – a “node set”. For a particular tier in the hierarchy, there might be x node sets. Each node set would have some number of genes and the total number of genes would be represented by the total node sets across the tier. For each node set, the average of the functional data for the member genes were averaged and compared with a bootstrap sample from the entire dataset. The data was represented graphically as a cluster heatmap, where each tier was a row in the graph, and a node set was a rectangle, colored by its average value. The purple colored tiles in Fig. 6E represent the absolute magnitude of the gene’s effects, regardless of direction. In this type of analysis, one might uncover groups of related genes that work together, both positively and negatively, to regulate cellular processes.

The uncorrected bootstrap statistics were indicated by “.” and “:” (0.05 and 0.01 respectively). Since a small family could be easily skewed by the presence of an outlier, the sample mean was also compared to a bootstrap spiked with the outlier value (first inverse jackknife, [78]), which was more stringent and eliminated families that were only skewed by one outlier. The bootstraps were also corrected, per tier by Benjamini/Hochberg [79] (alpha 0.05). The analyses were run separately for each parameter tested, and significant values were summarized. In Fig. 6E, tiles were shown with the same heatmap color as in the cluster heatmap, and were outlined if they had uncorrected p<0.01 or better (or either of the corrected p<0.05).

## Supporting Information

Figure S1 **DRG Enrichment in Subtraction Library Genes.** 27 Genes from the subtraction library were tested using Q-PCR. Samples of DRG and cerebellum mRNA were probed for the presence of the genes on the x-axis. Nine genes showed greater than two fold increases in DRGs, and seven of these were expressed well over 3 fold more in DRGs than cerebellum. Only one gene had over 2 fold expression in cerebellum, and the others were not differentially expressed.
(TIF)

Figure S2 **Genes in Screened Library Deficient in Cerebellar Expression.** Of the 1,100 genes in the screened library, over 800 are annotated in the Allen Brain Atlas (www.brain-map.org). Data about the in-situ expression pattern in adult C57/Bl6 mouse brains were extracted and compared. Ratios of each brain region’s expression density, compared to cerebellar expression density, was plotted for the screened library (filled bars) and a random sampling of genes from the Atlas (white bars). Error bars indicate 95% confidence intervals for the ratio. In the screened library, cerebellar expression was significantly reduced from the density in other brain regions, when compared with the random set, except for the olfactory bulb. Asterisk above bars indicate significance (*p<0.05, **p<0.01, ***p<0.001) from ANOVA. The label reading “hippocampus” is denoted “hippocampal formation” in the Allen Brain Atlas.
(TIF)

Figure S3 **CSPG Inhibition Robust Across Entire Screen, Independent of Basal Laminin Growth.** Individual experiments from the screen tested ∼80 genes each. Each was done on different days with different mice. Basal outgrowth on laminin varied from experiment to experiment, and had a wide range. Cells from individual gene transfections were plated onto both laminin and CSPG substrates. Keeping markers for laminin (black) and CSPG (white) as a vertical pair, the results were sorted on the x axis for laminin growth rank (longest neurites on the left  = 0, with the shortest  = 100). From this graph it is clear that growth was highly variable across experiments and conditions, and that normalization is necessary to extract meaningful data. It is also apparent that CSPG inhibition is robust across the full range of basal laminin growth and that some treatments were able to alter the CSPG growth towards or away from the basal CSPG level.
(TIF)

Figure S4 **Analysis of functional effects in gene families.** An example of the concept and construction of a tile cluster analysis using molecular function gene ontology (GO, http://amigo.geneontology.org/) annotations. ***A***, Table with 19 genes from the screened library and accompanying GO terms (gene has ontology annotation if cell is blue). ***B***, Genes are clustered using just their ontology annotations by hierarchical clustering, resulting in a dendrogram. Moving from the root of the dendrogram (left) towards the leaves (right) leads to more groups with fewer, more closely related genes in each group. ***C***, Bar chart depicting the functional data from the screen centered on the population mean. Bars have heatmap coloring, with genes decreasing growth in red, increasing growth in green, or having no effect compared to control in white (increasing effects coded by increasing color intensities). ***D***, Tile cluster analysis results – a multi-tiered heat map. The number of clusters per tier is listed and significance is calculated non-parametrically with bootstrap analysis, and is shown symbolically over the tile with an asterisk.
(TIF)

Figure S5 **Analyzed variables SSMD and PCA.** SSMD of the mCherry (negative control) vs. mCherry Gö6976 (positive control) for neurons growing on CSPGs (**A**), or Laminin (**B**). SSMD was calculated with the absolute/raw values (red bars), or the relative/normalized values (blue bars). Distinct groups are apparent, with neurite count and fraction of neurons with neurites parameters dominating the CSPG parameters. Principal component analysis (PCA) was performed and returned four components. Panel C plots the first two components with experimental plasmids (red markers) and control plasmids (blue markers), where the non-transfected controls are on the top left while positive controls are located on the bottom left. The PCA weights indicated clustering of the measured parameters such that like variables were combined (**D**).
(PNG)

Table S1 Myelin purified from P25-30 day old C57/Bl6j was dried down on poly-lysine 96-well plates. This inhibitory substrate was used to challenge transfected cerebellar neurons. Over 250 clones were screened due to their full-length status in the original library. The table shows aggregate results from approximately 12 experiments, where overlapping subsets of the clones were tested. The genes listed were observed to increase growth on myelin. *Anxa2 had the strongest effect in conjunction with forskolin (increase cAMP). More symbols indicate that the effect was observed in multiple experiments.
(DOC)

Table S2 Regeneration associated genes identified in the literature over the past 15 years and screened in our assay. The first six columns indicate effect on the parameters: 1,4, branches, 2,5 neurite average length, 3,6 number of primary neurites. Columns 1–3 indicate effects on CSPG substrate, and 4–6 indicate effects on laminin substrate. A “+” or “−” is listed if either: all the cells, the GFP+ cells, or the Neurite + cells were significantly above (“++” p<0.05 Mann Whitney U) or below (“–” p<0.05 Mann Whitney U) the control genes (see methods). Non-significant results are also listed, indicating the trend and direction of response (+, − p<0.1, Mann Whitney U). Blank cells indicate p> = 0.1. The official Entrez gene symbol is listed.
(DOC)

Table S3 **Confirmed Results after Secondary Screen.** Sixteen genes had significant effects compared to pSport mCherry control. The listed genes were significant for a particular parameter by Mann Whitney U compared to the control in up to four experimental replicates (++, −− p<0.05, +++, −−− p<0.01). N+ is percent of neurite initiation, BPTC is total branches, G+ indicates using only GFP+ cells. NTC is the number of primary neurites, and NTL is the neurite total length.
(DOC)

Table S4 **SSMD for various measurements**. SSMDs for the normalized forms of several parameters recorded as part of the screen, listed both for CSPGs and Laminin, using pSmCherry as the negative and pSmCherry Gö6976 as the positive control. Column headers are a concatenation between the subset (first abbreviation; Ap  =  complete, Np  =  neurite+, Tp  =  transfected+) and variation (Second abbreviation; Av  =  average, Ln  =  log, and Sq  =  square).
(DOC)

Methods S1 Supplementary Methods.
(DOCX)
