# Supplementary figures and images for: Peripheral Nervous System Genes Expressed in Central Neurons Induce Growth on Inhibitory Substrates
Source: PLoS One. 2012 Jun 6;7(6):e38101. doi: 10.1371/journal.pone.0038101 (PMC3368946; doi:10.1371/journal.pone.0038101)

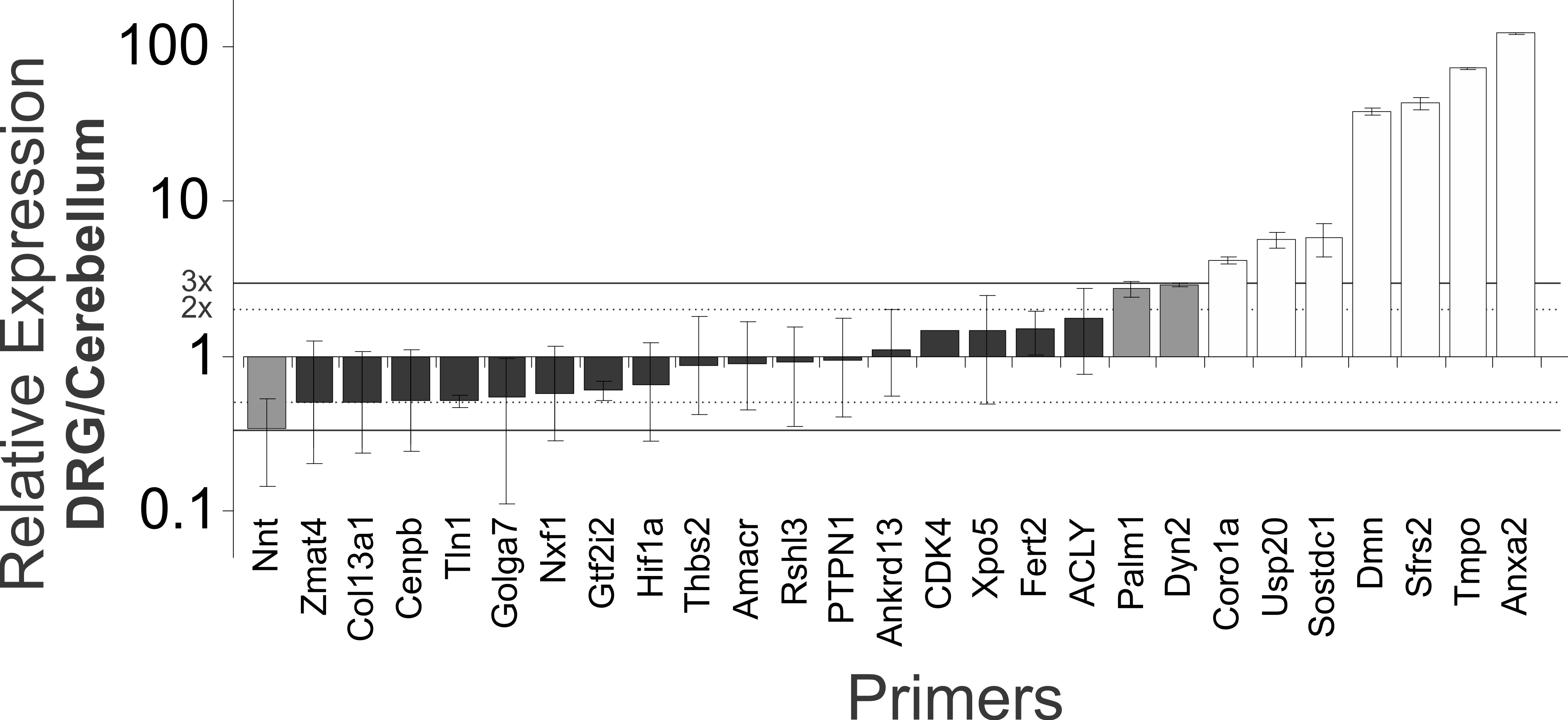

Supplement: Figure S1 — DRG Enrichment in Subtraction Library Genes. 27 Genes from the subtraction library were tested using Q-PCR. Samples of DRG and cerebellum mRNA were probed for the presence of the genes on the x-axis. Nine genes showed greater than two fold increases in DRGs, and seven of these were expressed well over 3 fold more in DRGs than cerebellum. Only one gene had over 2 fold expression in cerebellum, and the others were not differentially expressed. (TIF) [file pone.0038101.s001.tif]

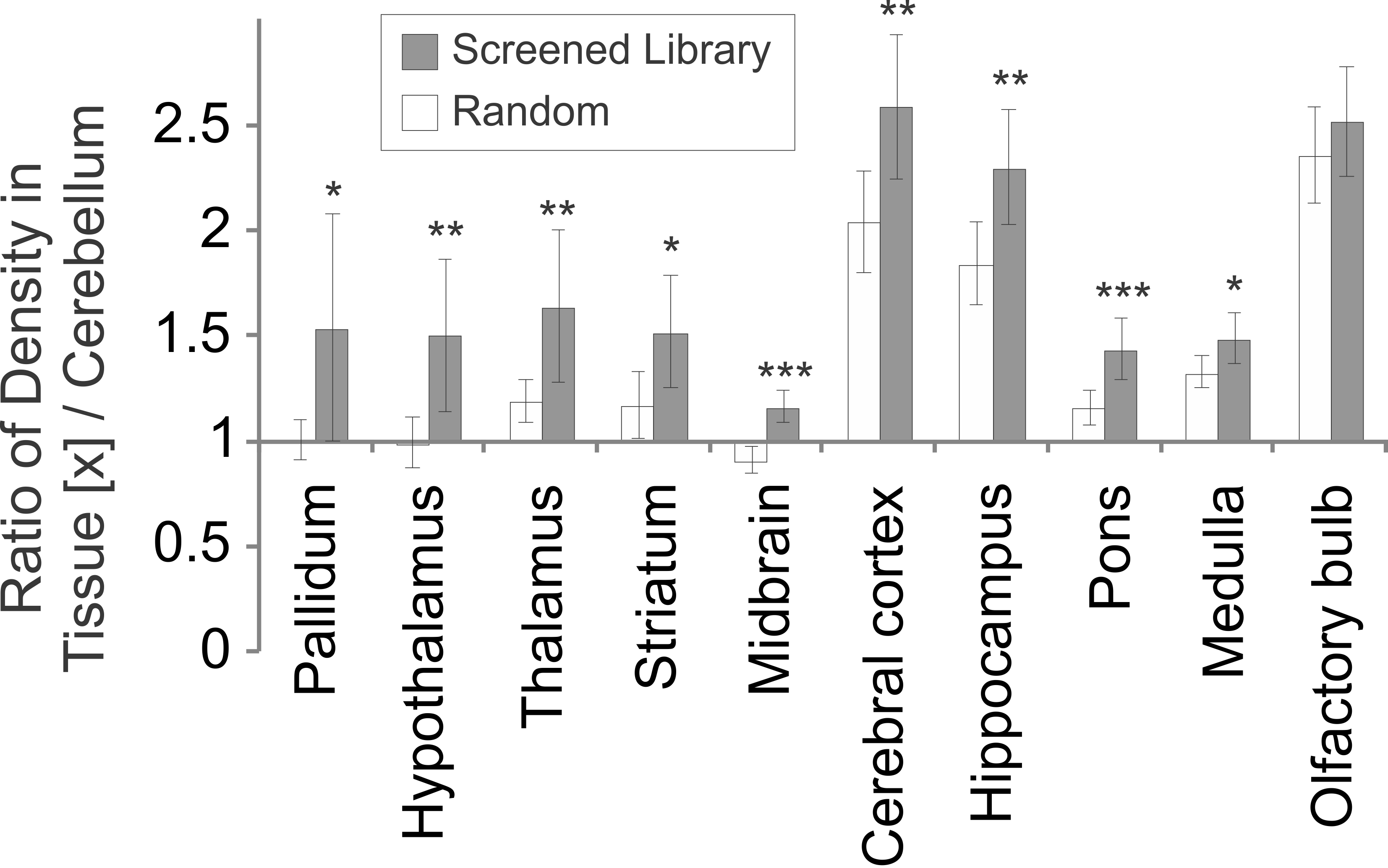

Supplement: Figure S2 — Genes in Screened Library Deficient in Cerebellar Expression. Of the 1,100 genes in the screened library, over 800 are annotated in the Allen Brain Atlas (www.brain-map.org). Data about the in-situ expression pattern in adult C57/Bl6 mouse brains were extracted and compared. Ratios of each brain region’s expression density, compared to cerebellar expression density, was plotted for the screened library (filled bars) and a random sampling of genes from the Atlas (white bars). Error bars indicate 95% confidence intervals for the ratio. In the screened library, cerebellar expression was significantly reduced from the density in other brain regions, when compared with the random set, except for the olfactory bulb. Asterisk above bars indicate significance (*p<0.05, **p<0.01, ***p<0.001) from ANOVA. The label reading “hippocampus” is denoted “hippocampal formation” in the Allen Brain Atlas. (TIF) [file pone.0038101.s002.tif]

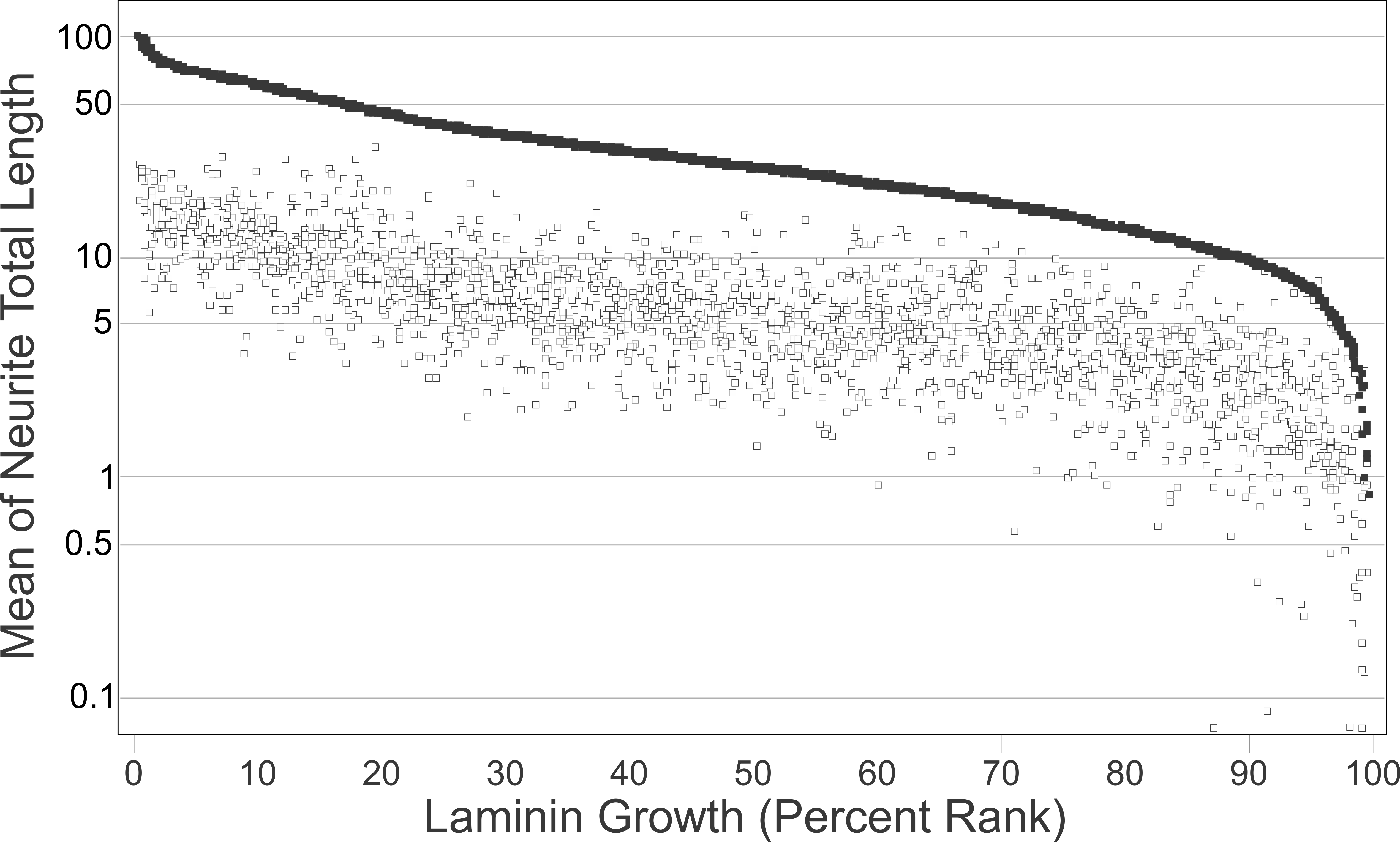

Supplement: Figure S3 — CSPG Inhibition Robust Across Entire Screen, Independent of Basal Laminin Growth. Individual experiments from the screen tested ∼80 genes each. Each was done on different days with different mice. Basal outgrowth on laminin varied from experiment to experiment, and had a wide range. Cells from individual gene transfections were plated onto both laminin and CSPG substrates. Keeping markers for laminin (black) and CSPG (white) as a vertical pair, the results were sorted on the x axis for laminin growth rank (longest neurites on the left = 0, with the shortest = 100). From this graph it is clear that growth was highly variable across experiments and conditions, and that normalization is necessary to extract meaningful data. It is also apparent that CSPG inhibition is robust across the full range of basal laminin growth and that some treatments were able to alter the CSPG growth towards or away from the basal CSPG level. (TIF) [file pone.0038101.s003.tif]

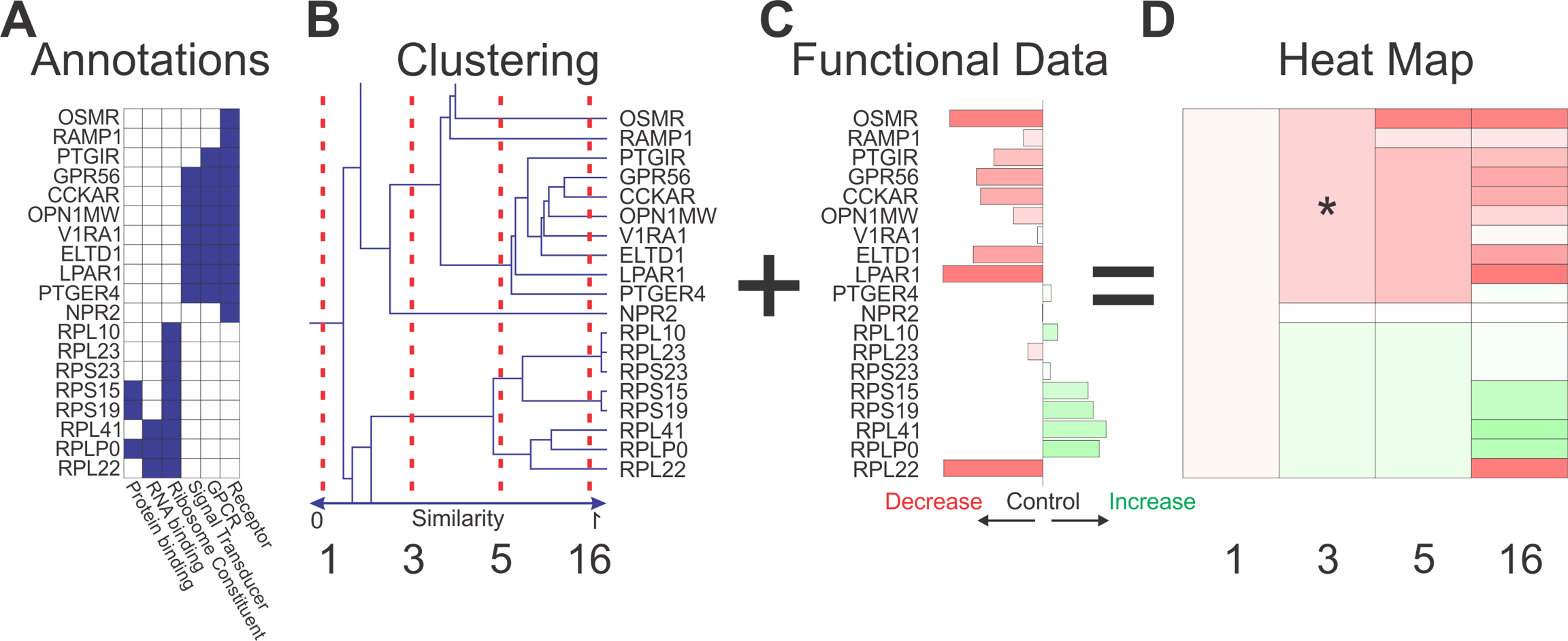

Supplement: Figure S4 — Analysis of functional effects in gene families. An example of the concept and construction of a tile cluster analysis using molecular function gene ontology (GO, http://amigo.geneontology.org/) annotations. A, Table with 19 genes from the screened library and accompanying GO terms (gene has ontology annotation if cell is blue). B, Genes are clustered using just their ontology annotations by hierarchical clustering, resulting in a dendrogram. Moving from the root of the dendrogram (left) towards the leaves (right) leads to more groups with fewer, more closely related genes in each group. C, Bar chart depicting the functional data from the screen centered on the population mean. Bars have heatmap coloring, with genes decreasing growth in red, increasing growth in green, or having no effect compared to control in white (increasing effects coded by increasing color intensities). D, Tile cluster analysis results – a multi-tiered heat map. The number of clusters per tier is listed and significance is calculated non-parametrically with bootstrap analysis, and is shown symbolically over the tile with an asterisk. (TIF) [file pone.0038101.s004.tif]

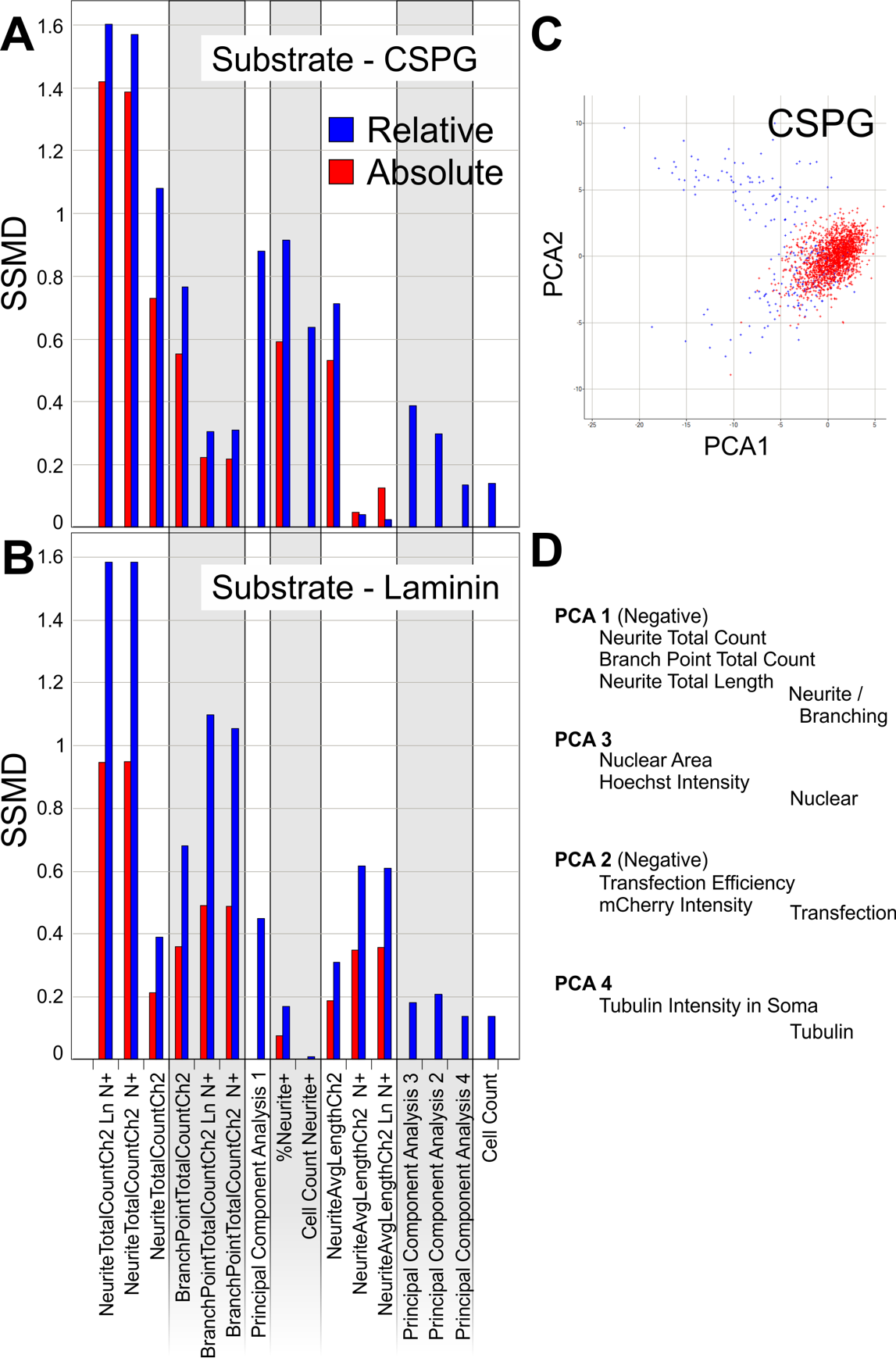

Supplement: Figure S5 — Analyzed variables SSMD and PCA. SSMD of the mCherry (negative control) vs. mCherry Gö6976 (positive control) for neurons growing on CSPGs (A), or Laminin (B). SSMD was calculated with the absolute/raw values (red bars), or the relative/normalized values (blue bars). Distinct groups are apparent, with neurite count and fraction of neurons with neurites parameters dominating the CSPG parameters. Principal component analysis (PCA) was performed and returned four components. Panel C plots the first two components with experimental plasmids (red markers) and control plasmids (blue markers), where the non-transfected controls are on the top left while positive controls are located on the bottom left. The PCA weights indicated clustering of the measured parameters such that like variables were combined (D). (PNG) [file pone.0038101.s005.png]
