# Supplementary material for: Peripheral Nervous System Genes Expressed in Central Neurons Induce Growth on Inhibitory Substrates
Source: PLoS One. 2012 Jun 6;7(6):e38101. doi: 10.1371/journal.pone.0038101 (PMC3368946; doi:10.1371/journal.pone.0038101)
Supplement: Table S1 — Myelin purified from P25-30 day old C57/Bl6j was dried down on poly-lysine 96-well plates. This inhibitory substrate was used to challenge transfected cerebellar neurons. Over 250 clones were screened due to their full-length status in the original library. The table shows aggregate results from approximately 12 experiments, where overlapping subsets of the clones were tested. The genes listed were observed to increase growth on myelin. *Anxa2 had the strongest effect in conjunction with forskolin (increase cAMP). More symbols indicate that the effect was observed in multiple experiments. (DOC) [file pone.0038101.s006.doc]

### Supplemental Table 1. Genes of interest from preliminary myelin screen.

| Symbol | Name | Effect |
| --- | --- | --- |
| DMN | Desmuslin | ●●●● |
| SOSTDC1 | Sclerostin Domain Containing 1 | ●●●● |
| ANXA2 | Annexin A2* | ●●●● |
| THBS2 | Thrombospondin 2 | ●●● |
| ACLY | ATP Citrate Lyase | ●● |
| CDK4 | Cyclin Dependent Kinase 4 | ●● |
| SFRS2 | Splicing Factor SC-35 | ●● |
| ANKRD13 | Ankyrin Repeat Domain 13A | ●● |
| TIRAP | Toll-interleukin 1 receptor domain containing adaptor | ● |
| PTPN1 | Protein tyrosine phosphatases, non-receptor type 1 | ● |

**Supplemental Table 1**. Myelin purified from P25-30 day old C57/Bl6j was dried down on poly-lysine 96-well plates. This inhibitory substrate was used to challenge transfected cerebellar neurons. Over 250 clones were screened due to their full-length status in the original library. The table shows aggregate results from approximately 12 experiments, where overlapping subsets of the clones were tested. The genes listed were observed to increase growth on myelin. *Anxa2 had the strongest effect in conjunction with forskolin (increase cAMP). More symbols indicate that the effect was observed in multiple experiments.
