# Supplementary material for: Peripheral Nervous System Genes Expressed in Central Neurons Induce Growth on Inhibitory Substrates
Source: PLoS One. 2012 Jun 6;7(6):e38101. doi: 10.1371/journal.pone.0038101 (PMC3368946; doi:10.1371/journal.pone.0038101)
Supplement: Table S2 — Regeneration associated genes identified in the literature over the past 15 years and screened in our assay. The first six columns indicate effect on the parameters: 1,4, branches, 2,5 neurite average length, 3,6 number of primary neurites. Columns 1–3 indicate effects on CSPG substrate, and 4–6 indicate effects on laminin substrate. A “+” or “−” is listed if either: all the cells, the GFP+ cells, or the Neurite + cells were significantly above (“++” p<0.05 Mann Whitney U) or below (“–” p<0.05 Mann Whitney U) the control genes (see methods). Non-significant results are also listed, indicating the trend and direction of response (+, − p<0.1, Mann Whitney U). Blank cells indicate p> = 0.1. The official Entrez gene symbol is listed. (DOC) [file pone.0038101.s007.doc]

### Supplemental Table 2. Performance of known neuronal growth regulating genes in primary screen.

| 1 | 2 | 3 | 4 | 5 | 6 | Gene Symbol | Common Name | Note | Citation |
| --- | --- | --- | --- | --- | --- | --- | --- | --- | --- |
|  |  |  |  |  |  | APBB1 | Amyloid beta (A4) | Inhibits axon branching | (Ikin et al., 2007) |
| - | - | - | + |  | - | ARC | Arc | Stimulates extension in neuroblastoma | (Donai et al., 2003) |
| + | ++ | ++ |  |  |  | BDNF | BDNF | Dose dependent effect | (Schecterson and Bothwell, 1992) |
|  |  |  |  |  |  | CD24 | CD24 | Interacts with L1, Inhibits neurite growth. | (Shewan et al., 1996) |
|  |  |  |  |  |  | CDK5R1 | p35 | Inhibits PAK | (Nikolic et al., 1998) |
| -- | -- | -- |  | -- | -- | CDKN2A | p21 | Sufficient to rescue PC12 cell differentiation | (Poluha et al., 1997)(Daniels et al., 1998)(Daniels et al., 1998) |
|  |  |  |  |  |  | CFL1 | Cofilin 1 | Effector of neurite extension | (Meberg et al., 1998) |
|  | + | ++ |  | ++ |  | CSK | c-Src | Inhibits neurite growth in PC12 | (Dey et al., 2005) |
| -- | -- | -- | -- | -- | -- | DUSP6 | MKP3 | Correlates with PC12 neuritogenesis | (Camps et al., 1998) |
| + |  | ++ |  |  |  | FGFR1 | FGF Receptor 1 | Overexpression promoted outgrowth | (Hausott et al., 2008) |
|  |  |  |  |  |  | FRK | Fyn related (GTK) | Induces NGF independent PC12 growth | (Anneren et al., 2000)(Tombran-Tink and Barnstable, 2003)(Tombran-Tink and Barnstable, 2003) |
| - | -- | -- | - |  |  | FYN | FYN | Fyn KO mice lack NCAM neurite growth | (Beggs et al., 1994) |
|  |  |  |  |  |  | GAL | Galanin | Re-expressed after injury | (Villar et al., 1989) |
|  |  | ++ |  |  |  | GSK3A | GSK3 a | Has distinct function from GSK3B | (Yoshimura et al., 2005; Lee et al., 2007) |
| -- |  | - |  |  |  | IGF1R | IGF1 Receptor | Receptor is essential for Hippocampal polarity | (Sosa et al., 2006) |
|  |  |  |  |  |  | ITGA3 | Integrin alpha 3 | Mediates outgrowth | (Defreitas et al., 1995) |
| - | -- | - |  | -- | -- | JUN | Jun | Required for axon regeneration | (Herdegen et al., 1997; Raivich et al., 2004) |
|  |  |  |  |  |  | LDLR | LDL receptor | Promotes Apolipo Protein E Dependent growth | (Holtzman et al., 1995) |
| - | - |  |  |  |  | LIFR | LIF receptor | Inhibits growth in sympathetic neurons | (Ng et al., 2003) |
|  |  |  |  | -- | -- | LIMK2 | Lim kinase | Balances with Slingshot to control ADF/cofilin | (Meberg et al., 1998) |
|  |  |  |  |  |  | MMP9 | MMP9 | MMP9 deficiency affects neurite outgrowth | (Vaillant et al., 2003) |
|  |  |  |  |  |  | NPTX2 | Neuronal pentraxin | Promotes growth | (Tsui et al., 1996) |
| + | ++ | ++ |  |  |  | PDGFRB | PDGF Receptor | Neuritogenesis in SH-SY5Y cells | (Hynds et al., 1995) |
| - |  |  |  |  |  | PIK3R4 | PIK3 | PI3K inhibitors reduce axon elongation | (Menager et al., 2004) |
|  | -- | - |  |  |  | PRKACA | PKA Catalytic | Inhibit PKA stops forskolin growth | (Chijiwa et al., 1990) |
|  |  |  |  |  |  | PTEN | PTEN | PTEN KO retinal regrowth after crush | (Park et al., 2008) |
|  | -- | -- |  |  |  | PTPN1 | PTP-1b | Regulates neurite extension by CAMs | (Pathre et al., 2001) |
| - |  |  |  |  | - | RARA | Retinoic acid Rcp. | Beta 2 promotes sensory axon recovery in SC | (Wong et al., 2006) |
|  |  |  | ++ | + | ++ | RELA | Rela/p65, NFkB | Status determines promotion/inhibition of growth | (Gutierrez et al., 2008) |
| - | -- |  |  |  | - | RHOB | Rho B | Expressed during lack of growth | (Brabeck et al., 2004) |
|  |  | ++ | + |  |  | RORA | RAR Orphan Rcp. | Overexpression protects against stress apoptosis | (Boukhtouche et al., 2006) |
|  | -- | -- |  |  | - | SERPINF1 | PEDF | Neuro-protective when added as protein | (Tombran-Tink and Barnstable, 2003)(Tombran-Tink and Barnstable, 2003; Tombran-Tink and Barnstable, 2003; Yabe et al., 2001; Araki et al., 1998; Tombrantink et al., 1991)(Tombran-Tink and Barnstable, 2003; Tombran-Tink and Barnstable, 2003; Yabe et al., 2001; Araki et al., 1998; Tombrantink et al., 1991) |
|  |  |  |  | - | - | SMURF1 | SMAD specific E3 | Ubiquitinates Rhoa, and promotes growth | (Vohra et al., 2007; Bryan et al., 2005) |
| - | -- | -- |  |  |  | SOCS6 | Socs6 | Socs2 induces outgrowth through EGFR | (Goldshmit et al., 2004) |
| + |  |  |  |  |  | SPP1 | Osteopontin | Inhibits Neurite Outgrowth | (Kury et al., 2005) |
|  | + |  |  |  |  | STAT3 | Stat3 | IL6 > Stat3 > PC12 cell growth | (Wu and Bradshaw, 1996)(Bradshaw et al., 1994)(Bradshaw et al., 1994) |
|  |  |  |  |  |  | TMPO | Thymopoietin | Induces process formation in PC12 cells | (Quik et al., 1990) |

**Supplementary Table S2.** Regeneration associated genes identified in the literature over the past 15 years and screened in our assay. The first six columns indicate effect on the parameters: 1,4, branches, 2,5 neurite average length, 3,6 number of primary neurites. Columns 1-3 indicate effects on CSPG substrate, and 4-6 indicate effects on laminin substrate. A “+” or “-” is listed if either: all the cells, the GFP+ cells, or the Neurite + cells were significantly above (“++” p < 0.05 Mann Whitney U) or below (“--" p < 0.05 Mann Whitney U) the control genes (see methods). Non-significant results are also listed, indicating the trend and direction of response (+,- p < 0.1, Mann Whitney U). Blank cells indicate p>=0.1. The official Entrez gene symbol is listed.

## REFERENCES

Anneren C, Reedquist KA, Bos JL,Welsh M (2000) GTK, a Src-related tyrosine kinase, induces nerve growth factor-independent neurite outgrowth in PC12 cells through activation of the Rap1 pathway. Relationship to Shb tyrosine phosphorylation and elevated levels of focal adhesion kinase. J Biol Chem 275:29153-29161.

Araki T, Taniwaki T, Becerra SP, Chader GJ,Schwartz JP (1998) Pigment epithelium-derived factor (PEDF) differentially protects immature but not mature cerebellar granule cells against apoptotic cell death. J Neurosci Res 53:7-15.

Ashburner M, Ball CA, Blake JA, Botstein D, Butler H, Cherry JM, Davis AP, Dolinski K, Dwight SS, Eppig JT, Harris MA, Hill DP, Issel-Tarver L, Kasarskis A, Lewis S, Matese JC, Richardson JE, Ringwald M, Rubin GM,Sherlock G (2000) Gene ontology: tool for the unification of biology. The Gene Ontology Consortium. Nat Genet 25:25-29.

Beggs HE, Soriano P,Maness PF (1994) NCAM-dependent neurite outgrowth is inhibited in neurons from Fyn-minus mice. J Cell Biol 127:825-833.

Boukhtouche F, Vodjdani G, Jarvis CI, Bakouche J, Staels B, Mallet J, Mariani J, Lemaigre-Dubreuil Y,Brugg B (2006) Human retinoic acid receptor-related orphan receptor alpha1 overexpression protects neurones against oxidative stress-induced apoptosis. J Neurochem 96:1778-1789.

Brabeck C, Beschorner R, Conrad S, Mittelbronn M, Bekure K, Meyermann R, Schluesener HJ,Schwab JM (2004) Lesional expression of RhoA and RhoB following traumatic brain injury in humans. J Neurotrauma 21:697-706.

Bradshaw RH, Bubier NE,Sullivan M (1994) The Effects of Age and Gender on Perceived Facial Attractiveness - a Reply. Canadian Journal of Behavioural Science-Revue Canadienne Des Sciences Du Comportement 26:199-204.

Bryan B, Cai Y, Wrighton K, Wu G, Feng XH,Liu MY (2005) Ubiquitination of RhoA by Smurf1 promotes neurite outgrowth. FEBS Lett 579:1015-1019.

Camps M, Chabert C, Muda M, Boschert U, Gillieron C,Arkinstall S (1998) Induction of the mitogen-activated protein kinase phosphatase MKP3 by nerve growth factor in differentiating PC12. FEBS Lett 425:271-276.

Chijiwa T, Mishima A, Hagiwara M, Sano M, Hayashi K, Inoue T, Naito K, Toshioka T,Hidaka H (1990) Inhibition of forskolin-induced neurite outgrowth and protein phosphorylation by a newly synthesized selective inhibitor of cyclic AMP-dependent protein kinase, N-[2-(p-bromocinnamylamino)ethyl]-5-isoquinolinesulfonamide (H-89), of PC12D pheochromocytoma cells. J Biol Chem 265:5267-5272.

Daniels RH, Hall PS,Bokoch GM (1998) Membrane targeting of p21-activated kinase 1 (PAK1) induces neurite outgrowth from PC12 cells. EMBO J 17:754-764.

Defreitas MF, Yoshida CK, Frazier WA, Mendrick DL, Kypta RM,Reichardt LF (1995) Identification of Integrin Alpha(3)beta-1 as a Neuronal Thrombospondin Receptor Mediating Neurite Outgrowth. Neuron 15:333-343.

Dey N, Howell BW, De PK,Durden DL (2005) CSK negatively regulates nerve growth factor induced neural differentiation and augments AKT kinase activity. Exp Cell Res 307:1-14.

Donai H, Sugiura H, Ara D, Yoshimura Y, Yamagata K,Yamauchi T (2003) Interaction of Arc with CaM kinase II and stimulation of neurite extension by Arc in neuroblastoma cells expressing CaM kinase II. Neurosci Res 47:399-408.

Goldshmit Y, Walters CE, Scott HJ, Greenhalgh CJ,Turnley AM (2004) SOCS2 induces neurite outgrowth by regulation of epidermal growth factor receptor activation. J Biol Chem 279:16349-16355.

Gutierrez H, O'Keeffe GW, Gavalda N, Gallagher D,Davies AM (2008) Nuclear factor kappa B signaling either stimulates or inhibits neurite growth depending on the phosphorylation status of p65/RelA. J Neurosci 28:8246-8256.

Hausott B, Schlick B, Vallant N, Dorn R,Klimaschewski L (2008) Promotion of neurite outgrowth by fibroblast growth factor receptor 1 overexpression and lysosomal inhibition of receptor degradation in pheochromocytoma cells and adult sensory neurons. Neuroscience 153:461-473.

Herdegen T, Skene P,Bahr M (1997) The c-Jun transcription factor--bipotential mediator of neuronal death, survival and regeneration. Trends Neurosci 20:227-231.

Holtzman DM, Pitas RE, Kilbridge J, Nathan B, Mahley RW, Bu G,Schwartz AL (1995) Low density lipoprotein receptor-related protein mediates apolipoprotein E-dependent neurite outgrowth in a central nervous system-derived neuronal cell line. Proc Natl Acad Sci U S A 92:9480-9484.

Hynds DL, Summers M, Van Brocklyn J, O'Dorisio MS,Yates AJ (1995) Gangliosides inhibit platelet-derived growth factor-stimulated growth, receptor phosphorylation, and dimerization in neuroblastoma SH-SY5Y cells. J Neurochem 65:2251-2258.

Ikin AF, Sabo SL, Lanier LM,Buxbaum JD (2007) A macromolecular complex involving the amyloid precursor protein (APP) and the cytosolic adapter FE65 is a negative regulator of axon branching. Molecular and Cellular Neuroscience 35:57-63.

Kury P, Zickler P, Stoll G, Hartung HP,Jander S (2005) Osteopontin, a macrophage-derived matricellular glycoprotein, inhibits axon outgrowth. FASEB J 19:398-400.

Lee HC, Tsai JN, Liao PY, Tsai WY, Lin KY, Chuang CC, Sun CK, Chang WC,Tsai HJ (2007) Glycogen synthase kinase 3 alpha and 3 beta have distinct functions during cardiogenesis of zebrafish embryo. BMC Dev Biol 7:93.

Meberg PJ, Ono S, Minamide LS, Takahashi M,Bamburg JR (1998) Actin depolymerizing factor and cofilin phosphorylation dynamics: response to signals that regulate neurite extension. Cell Motil Cytoskeleton 39:172-190.

Menager C, Arimura N, Fukata Y,Kaibuchi K (2004) PIP3 is involved in neuronal polarization and axon formation. J Neurochem 89:109-118.

Ng YP, He W,Ip NY (2003) Leukemia inhibitory factor receptor signaling negatively modulates nerve growth factor-induced neurite outgrowth in PC12 cells and sympathetic neurons. J Biol Chem 278:38731-38739.

Nikolic M, Chou MM, Lu W, Mayer BJ,Tsai LH (1998) The p35/Cdk5 kinase is a neuron-specific Rac effector that inhibits Pak1 activity. Nature 395:194-198.

Park KK, Liu K, Hu Y, Smith PD, Wang C, Cai B, Xu B, Connolly L, Kramvis I, Sahin M,He Z (2008) Promoting axon regeneration in the adult CNS by modulation of the PTEN/mTOR pathway. Science 322:963-966.

Pathre P, Arregui C, Wampler T, Kue I, Leung TC, Lilien J,Balsamo J (2001) PTP1B regulates neurite extension mediated by cell-cell and cell-matrix adhesion molecules. J Neurosci Res 63:143-150.

Poluha W, Schonhoff CM, Harrington KS, Lachyankar MB, Crosbie NE, Bulseco DA,Ross AH (1997) A novel, nerve growth factor-activated pathway involving nitric oxide, p53, and p21WAF1 regulates neuronal differentiation of PC12 cells. J Biol Chem 272:24002-24007.

Quik M, Cohen R, Audhya T,Goldstein G (1990) Thymopoietin Interacts at the Alpha-Bungarotoxin Site of and Induces Process Formation in Pc12 Pheochromocytoma Cells. Neuroscience 39:139-150.

Raivich G, Bohatschek M, Da Costa C, Iwata O, Galiano M, Hristova M, Nateri AS, Makwana M, Riera-Sans L, Wolfer DP, Lipp HP, Aguzzi A, Wagner EF,Behrens A (2004) The AP-1 transcription factor c-Jun is required for efficient axonal regeneration. Neuron 43:57-67.

Schecterson LC,Bothwell M (1992) Novel roles for neurotrophins are suggested by BDNF and NT-3 mRNA expression in developing neurons. Neuron 9:449-463.

Shewan D, Calaora V, Nielsen P, Cohen J, Rougon G,Moreau H (1996) mCD24, a glycoprotein transiently expressed by neurons, is an inhibitor of neurite outgrowth. J Neurosci 16:2624-2634.

Sosa L, Dupraz S, Laurino L, Bollati F, Bisbal M, Caceres A, Pfenninger KH,Quiroga S (2006) IGF-1 receptor is essential for the establishment of hippocampal neuronal polarity. Nat Neurosci 9:993-995.

Tombran-Tink J,Barnstable CJ (2003) PEDF: A multifaceted neurotrophic factor. Nature Reviews Neuroscience 4:628-636.

Tombrantink J, Chader GG,Johnson LV (1991) Pedf - a Pigment Epithelium-Derived Factor with Potent Neuronal Differentiative Activity. Exp Eye Res 53:411-414.

Tsui CC, Copeland NG, Gilbert DJ, Jenkins NA, Barnes C,Worley PF (1996) Narp, a novel member of the pentraxin family, promotes neurite outgrowth and is dynamically regulated by neuronal activity. J Neurosci 16:2463-2478.

Vaillant C, Meissirel C, Mutin M, Belin MF, Lund LR,Thomasset N (2003) MMP-9 deficiency affects axonal outgrowth, migration, and apoptosis in the developing cerebellum. Mol Cell Neurosci 24:395-408.

Villar MJ, Cortes R, Theodorsson E, Wiesenfeldhallin Z, Schalling M, Fahrenkrug J, Emson PC,Hokfelt T (1989) Neuropeptide Expression in Rat Dorsal-Root Ganglion-Cells and Spinal-Cord After Peripheral-Nerve Injury with Special Reference to Galanin. Neuroscience 33:587-604.

Vohra BPS, Fu M,Heuckeroth RO (2007) Protein kinase c zeta and glycogen synthase kinase-3 beta control neuronal polarity in developing rodent enteric neurons, whereas SMAD specific E3 ubiquitin protein ligase 1 promotes neurite growth but does not influence polarity. Journal of Neuroscience 27:9458-9468.

Wong LF, Yip PK, Battaglia A, Grist J, Corcoran J, Maden M, Azzouz M, Kingsman SM, Kingsman AJ, Mazarakis ND,McMahon SB (2006) Retinoic acid receptor beta2 promotes functional regeneration of sensory axons in the spinal cord. Nat Neurosci 9:243-250.

Wu YY,Bradshaw RA (1996) Induction of neurite outgrowth by interleukin-6 is accompanied by activation of Stat3 signaling pathway in a variant PC12 cell (E2) line. J Biol Chem 271:13023-13032.

Yabe T, Wilson D,Schwartz JP (2001) NF kappa B activation is required for the neuroprotective effects of pigment epithelium-derived factor (PEDF) on cerebellar granule neurons. J Biol Chem 276:43313-43319.

Yoshimura T, Kawano Y, Arimura N, Kawabata S, Kikuchi A,Kaibuchi K (2005) GSK-3beta regulates phosphorylation of CRMP-2 a
