# Supplementary material for: Peripheral Nervous System Genes Expressed in Central Neurons Induce Growth on Inhibitory Substrates
Source: PLoS One. 2012 Jun 6;7(6):e38101. doi: 10.1371/journal.pone.0038101 (PMC3368946; doi:10.1371/journal.pone.0038101)
Supplement: Table S3 — Confirmed Results after Secondary Screen. Sixteen genes had significant effects compared to pSport mCherry control. The listed genes were significant for a particular parameter by Mann Whitney U compared to the control in up to four experimental replicates (++, −− p<0.05, +++, −−− p<0.01). N+ is percent of neurite initiation, BPTC is total branches, G+ indicates using only GFP+ cells. NTC is the number of primary neurites, and NTL is the neurite total length. (DOC) [file pone.0038101.s008.doc]

### Supplemental Table 3. Confirmed Results

| **CSPG** | | | | | | | **Laminin** | | | | | | |  |  |
| --- | --- | --- | --- | --- | --- | --- | --- | --- | --- | --- | --- | --- | --- | --- | --- |
| N+ | BPTC | BPTC G+ | NTC | NTC G+ | NTL | NTL G+ | N+ | BPTC | BPTC G+ | NTC | NTC G+ | NTL | NTL G+ | **Symbol** | **Name** |
| ++ |  |  | ++ | ++ | ++ |  |  |  |  |  |  |  | ++ | mCherry Go6976 | Positive Control |
|  | -- |  |  |  |  | -- |  |  |  |  |  |  |  | ANXA2 | Annexin A2 |
|  |  |  |  |  |  |  | --- | --- |  | --- |  | --- |  | DUS3L | Dihydrouridine synthase 3-like |
|  | +++ | +++ | --- |  |  | +++ |  |  |  |  |  |  |  | EIF2B5 | Eukaryotic translation initiation factor 2B5 |
|  | ++ | ++ | ++ | ++ |  |  |  |  |  |  |  |  |  | GPX3 | Glutathione peroxidase 3 |
|  |  |  |  |  |  |  |  |  | --- |  | --- | -- | -- | GRIK1 | Glutamate receptor ionotropic kainate 1 |
|  |  |  |  |  |  |  | +++ |  | -- |  |  |  |  | IGH-6 | Ig heavy chain V region VH558 A1/A4 precursor |
|  | ++ |  |  |  |  |  |  |  |  |  |  |  |  | IVNS1ABP | influenza virus NS1A binding protein |
|  |  |  |  |  |  |  | -- |  |  |  |  |  |  | PDGFRB | Platelet-derived growth factor receptor beta |
| --- |  |  |  |  |  |  |  |  |  |  |  |  |  | PLA2G12A | Phospholipase A2 group XIIA |
|  | +++ | +++ |  |  |  |  |  |  |  |  |  |  |  | PRPF19 | PRP19/PSO4 pre-mRNA processing factor 19 |
|  |  |  |  |  |  |  | ++ |  |  |  | +++ | ++ | ++ | RBMX | RNA binding motif protein X chromosome |
|  |  |  |  |  |  |  |  | --- | -- |  | --- |  | --- | SDPR | Serum deprivation response |
|  | --- |  |  |  | -- | -- |  |  |  |  |  |  |  | SMARCAL1 | SWI/SNF matrix assoc. actin-dep. reg. of chromatin |
| -- | -- |  |  |  | -- |  |  | --- | +++ |  | ++ |  |  | SRPRB | Signal recognition particle receptor B |
|  |  |  |  |  |  |  | +++ |  | ++ |  | --- | ++ |  | 144845 | Defined by BC008631 |

**Supplemental Table 3. Confirmed Results after Secondary Screen**. Sixteen genes had significant effects compared to pSport mCherry control. The listed genes were significant for a particular parameter by Mann Whitney U compared to the control in up to four experimental replicates (++,-- p < 0.05, +++,--- p < 0.01). N+ is percent of neurite initiation, BPTC is total branches, G+ indicates using only GFP+ cells. NTC is the number of primary neurites, and NTL is the neurite total length.
