# Supplementary material for: Peripheral Nervous System Genes Expressed in Central Neurons Induce Growth on Inhibitory Substrates
Source: PLoS One. 2012 Jun 6;7(6):e38101. doi: 10.1371/journal.pone.0038101 (PMC3368946; doi:10.1371/journal.pone.0038101)
Supplement: Table S4 — SSMD for various measurements. SSMDs for the normalized forms of several parameters recorded as part of the screen, listed both for CSPGs and Laminin, using pSmCherry as the negative and pSmCherry Gö6976 as the positive control. Column headers are a concatenation between the subset (first abbreviation; Ap = complete, Np = neurite+, Tp = transfected+) and variation (Second abbreviation; Av = average, Ln = log, and Sq = square). (DOC) [file pone.0038101.s009.doc]

### Supplemental Table 4. SSMD

| **Abs SSMD** | **Ap_Av** | **Ap_Ln** | **Ap_Sq** | **Np_Av** | **Np_Ln** | **Np_Sq** | **Tp_Av** | **Tp_Ln** | **Tp_Sq** |
| --- | --- | --- | --- | --- | --- | --- | --- | --- | --- |
| **CSPGs** |  |  |  |  |  |  |  |  |  |
| AvgIntenCh3 Adj Log | 0.349 | 0.335 | 0.47 | 0.257 | 0.254 | 0.226 | 0.486 | 0.495 | 0.369 |
| BranchPointTotalCountCh2 | 0.766 | 0.778 | 0.474 | 0.31 | 0.306 | 0.173 | 0.578 | 0.583 | 0.516 |
| CellBodyAreaCh2 | 0.619 | 0.596 | 0.622 | 1.055 | 1.175 | 0.859 | 0.528 | 0.581 | 0.466 |
| CellBodyAvgIntenCh2 | 0.164 | 0.139 | 0.177 | 0.484 | 0.469 | 0.488 | 0.126 | 0.122 | 0.115 |
| CellBodyNucAvgIntenCh1 | 0.288 | 0.29 | 0.289 | 0.276 | 0.27 | 0.285 | 0.325 | 0.328 | 0.321 |
| CellBodyNucTotalAreaCh1 | 0.095 | 0.02 | 0.14 | 0.031 | 0.12 | 0.021 | 0.17 | 0.096 | 0.137 |
| GFP+ | 0.396 | 0.396 | 0.396 | 0.389 | 0.389 | 0.389 |  |  |  |
| NeuriteAvgLengthCh2 | 0.711 | 0.833 | 0.531 | 0.042 | 0.027 | 0.005 | 0.539 | 0.792 | 0.283 |
| NeuriteTotalCountCh2 | 1.08 | 1.066 | 1.16 | 1.568 | 1.602 | 1.371 | 1.174 | 1.16 | 1.19 |
| %GFP+ | 0.396 |  |  | 0.389 |  |  |  |  |  |
| %Neurite+ | 0.916 |  |  |  |  |  |  |  |  |
| Cell Count | 0.141 |  |  | 0.638 |  |  |  |  |  |
| NeuronNucleusRatio | 0.285 |  |  |  |  |  |  |  |  |
| PCA 1 | 0.88 |  |  |  |  |  |  |  |  |
| PCA 2 | 0.299 |  |  |  |  |  |  |  |  |
| PCA 3 | 0.388 |  |  |  |  |  |  |  |  |
| PCA 4 | 0.135 |  |  |  |  |  |  |  |  |
| **Laminin** |  |  |  |  |  |  |  |  |  |
| AvgIntenCh3 Adj Log | 0.211 | 0.199 | 0.368 | 0.453 | 0.446 | 0.542 | 0.366 | 0.37 | 0.3 |
| BranchPointTotalCountCh2 | 0.681 | 0.679 | 0.573 | 1.054 | 1.099 | 0.713 | 0.936 | 0.942 | 0.705 |
| CellBodyAreaCh2 | 0.155 | 0.14 | 0.163 | 0.262 | 0.25 | 0.265 | 0.035 | 0.034 | 0.038 |
| CellBodyAvgIntenCh2 | 0.112 | 0.208 | 0.048 | 0.025 | 0.109 | 0.031 | 0.072 | 0.094 | 0.061 |
| CellBodyNucAvgIntenCh1 | 0.048 | 0.09 | 0.015 | 0.081 | 0.12 | 0.046 | 0.009 | 0.043 | 0.017 |
| CellBodyNucTotalAreaCh1 | 0.128 | 0.128 | 0.098 | 0.272 | 0.249 | 0.256 | 0.09 | 0.056 | 0.105 |
| GFP+ | 0.272 | 0.272 | 0.272 | 0.523 | 0.523 | 0.523 |  |  |  |
| NeuriteAvgLengthCh2 | 0.311 | 0.237 | 0.335 | 0.618 | 0.61 | 0.473 | 0.534 | 0.534 | 0.393 |
| NeuriteTotalCountCh2 | 0.391 | 0.367 | 0.57 | 1.585 | 1.583 | 1.518 | 0.817 | 0.787 | 0.965 |
| %GFP+ | 0.272 |  |  | 0.523 |  |  |  |  |  |
| %Neurite+ | 0.17 |  |  |  |  |  |  |  |  |
| Cell Count | 0.139 |  |  | 0.012 |  |  |  |  |  |
| NeuronNucleusRatio | 0.107 |  |  |  |  |  |  |  |  |
| PCA1 | 0.449 |  |  |  |  |  |  |  |  |
| PCA 2 | 0.209 |  |  |  |  |  |  |  |  |
| PCA 3 | 0.184 |  |  |  |  |  |  |  |  |
| PCA 4 | 0.14 |  |  |  |  |  |  |  |  |

**Supplemental Table S4. SSMD for various measurements**. SSMDs for the normalized forms of several parameters recorded as part of the screen, listed both for CSPGs and Laminin, using pSmCherry as the negative and pSmCherry Gö6976 as the positive control. Column headers are a concatenation between the subset (first abbreviation; Ap = complete, Np = neurite+, Tp = transfected+) and variation (Second abbreviation; Av = average, Ln = log, and Sq = square).
