# Supplementary material for: Peripheral Nervous System Genes Expressed in Central Neurons Induce Growth on Inhibitory Substrates
Source: PLoS One. 2012 Jun 6;7(6):e38101. doi: 10.1371/journal.pone.0038101 (PMC3368946; doi:10.1371/journal.pone.0038101)
Supplement: Methods S1 — Supplementary Methods. (DOCX) [file pone.0038101.s010.docx]

## Supplementary Methods

### SSMDs

SMDs, or Strictly standardized mean differences, were used to establish the overall quality and fidelity of a particular parameter, in terms of its ability to segregate the negative (pSport mCherry) and the positive (pSport mCherry Gö6976) control. SSMD was estimated by (µ_neg_- µ_pos_)/√(σ_neg_^2^ + σ_pos_^2^). Since different variables were expected to report the positive and negative controls in opposite directions, the absolute was taken so that the effect size could be observed.

### False Discovery and False Negative Rates

False discovery rates (FDR) and false negative rates (FNR) were estimated by comparing positive and negative controls for the parameters rBPTC (branch point total count), rNAL (neurite average length), rNTC (number of primary neurites), and rfN (frequency of neurite initiation). The basic FDR was given by b/(b+d), and the FNR by c/(c+d), where b = false positive, c = false negative, d = true positives. The basic methodology used was reported previously (Zhang XD, et al. 2010). To estimate these values, a set of 166 positive and negative controls were used. Thresholds for the four stated variables were scanned by rank and the resulting “positive” and “negative” samples were counted, and used to generate the number of false positives, false negatives, and true positives. Then, finding a minimum where both the FDR and the FNR could be minimized, we calculated the FDR for parameters measured when neurons were cultured on CSPGs = rBPTC 31%, rNAL 33%, rNTC 24%, rfN 25%.

**Reference**

Zhang XD, Lacson R, Yang R, Marine SD, McCampbell A, Toolan DM, Hare TR, Kajdas J, Berger JP, Holder DJ, Heyse JF, Ferrer M (2010). The use of SSMD-based false discovery and false nondiscovery rates in genome-scale RNAi screens. J Biomol Screen. Oct;15(9):1123-31.
